# Supplementary material for: Mycobacterium tuberculosis associated with severe tuberculosis evades cytosolic surveillance systems and modulates IL-1β production
Source: Nat Commun. 2020 Apr 23;11:1949. doi: 10.1038/s41467-020-15832-6 (PMC7181847; doi:10.1038/s41467-020-15832-6)
Supplement: Supplementary file 1 — Supplementary Information [file 41467_2020_15832_MOESM1_ESM.pdf]

Mycobacterium tuberculosis associated with severe tuberculosis evades cytosolic surveillance systems and modulates IL-1β production

Sousa et al.

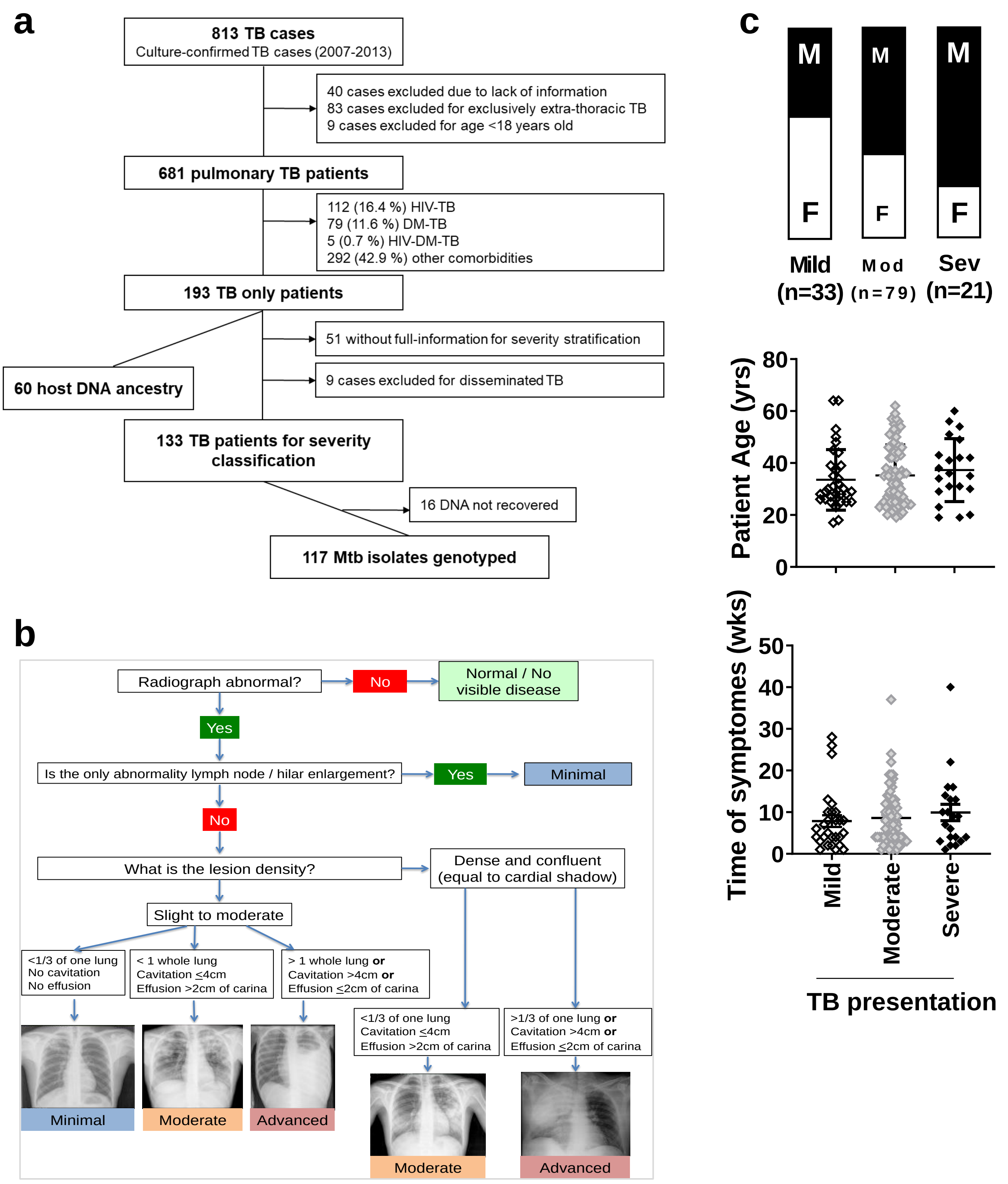

Supplementary Figure 1: Overview of the study population and characteristics of the selected TB patients. (A) Overview of the study population included in the different phases of the work. (B) Schematic representation of the chest X-ray classification method based on lymph node/ hilar abnormality and lesion density. (C) Distribution of gender (male, M; female, F), age and time of symptoms in mild, moderate and severe TB groups. In (C) Mean±SEM is represented.

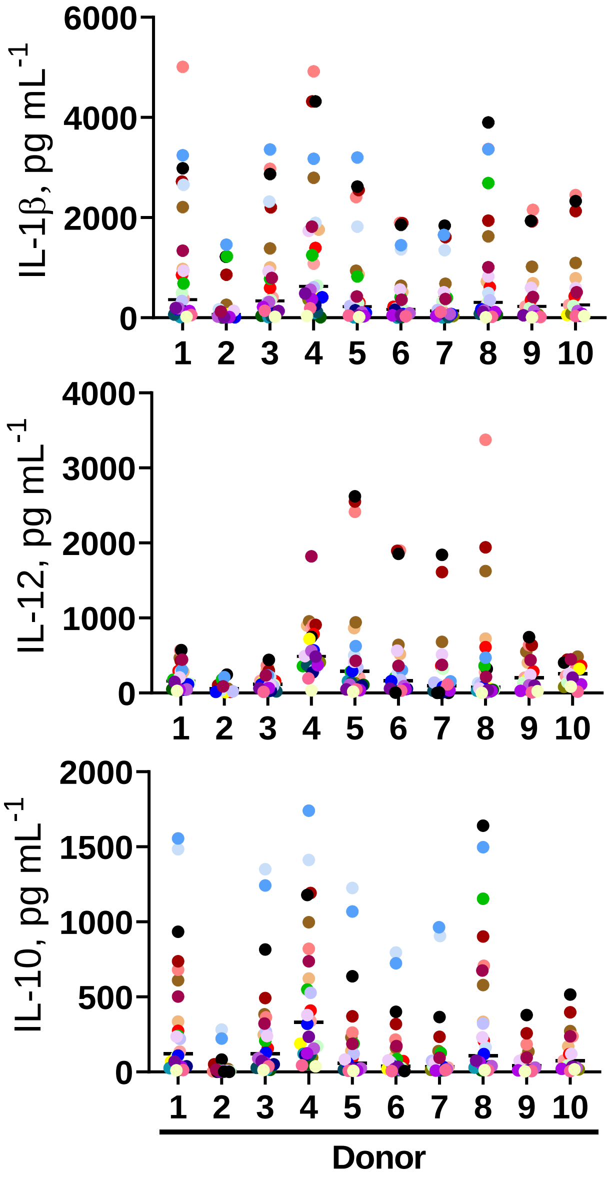

Supplementary Figure 2: Highly related *M. tuberculosis* isolates modulate cytokine production by infected PBMCs. Cytokine production by PBMCs infected for 24h with the 26 selected clinical *M. tuberculosis* isolates. Each number represents one donor and each dot one isolate. The same isolate is represented with the same color across the different donors. An MOI of 1 was used for infections.

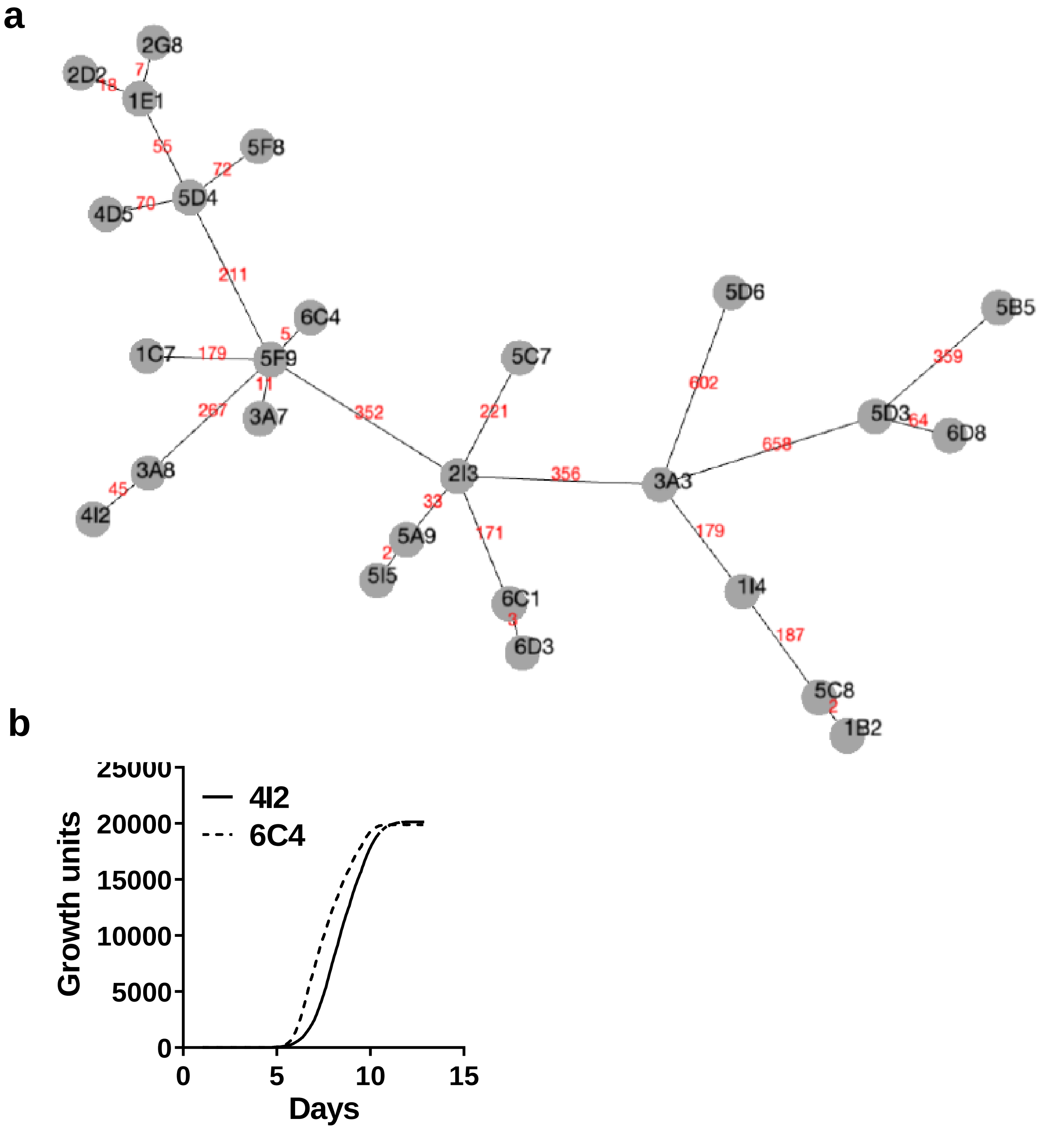

Supplementary Figure 3: Characterization of the selected *M. tuberculosis* isolates. (A) Possible transmission clusters identified among the 26 selected *M. tuberculosis* isolates. Minimum Spanning Tree used for visualizing the possible evolutionary relationships and number of pairwise SNPs (in red) between the studied clinical isolates. Clinical isolates separated by less than 12 SNPs were considered part of transmission clusters or pairs. (B) Growth profiles for the selected *M. tuberculosis* isolates 4I2 and 6C4. *M. tuberculosis* isolates 4I2 (solid line) and 6C4 (dashed line) were grown from standardized inocula in a Bactec MGIT 960. The growth units are plotted versus time (days).

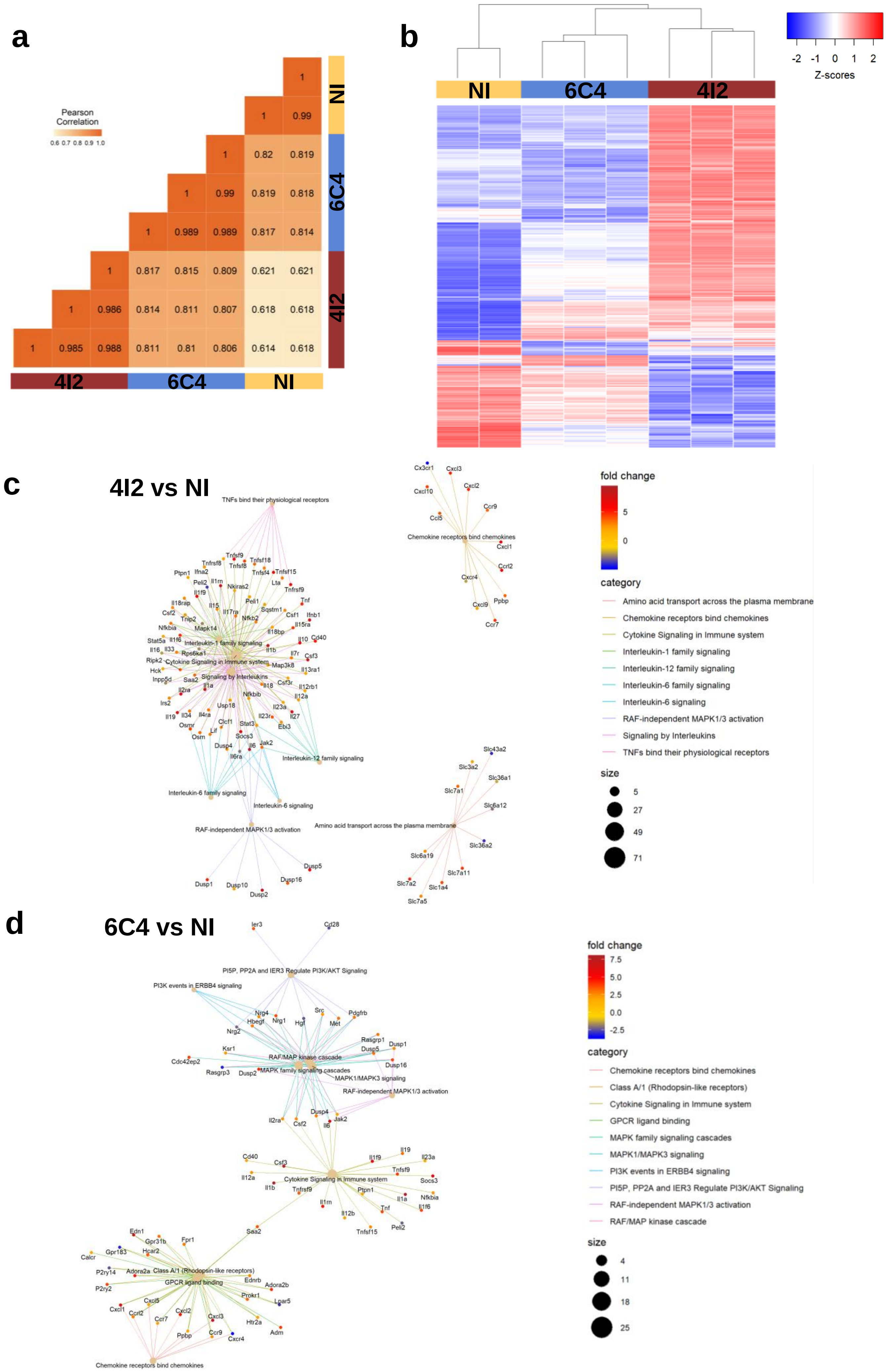

a
Pearson Correlation
0.6 0.7 0.8 0.9 1.0
1
1 0.99
1 0.82 0.819
1 0.99 0.819 0.818
1 0.989 0.989 0.817 0.814
1 0.817 0.815 0.809 0.621 0.621
1 0.986 0.814 0.811 0.807 0.618 0.618
1 0.985 0.988 0.811 0.81 0.806 0.614 0.618
NI
6C4
4I2
4I2
6C4
NI
b
-2 -1 0 1 2
Z-scores
NI
6C4
4I2
c
4I2 vs NI
TNFs bind their physiological receptors
Interleukin-1 family signaling
Cytokine Signaling in Immune system
Signaling by Interleukins
Interleukin-12 family signaling
Interleukin-6 family signaling
Interleukin-6 signaling
RAF-independent MAPK1/3 activation
Chemokine receptors bind chemokines
Amino acid transport across the plasma membrane
fold change
5
0
category
Amino acid transport across the plasma membrane
Chemokine receptors bind chemokines
Cytokine Signaling in Immune system
Interleukin-1 family signaling
Interleukin-12 family signaling
Interleukin-6 family signaling
Interleukin-6 signaling
RAF-independent MAPK1/3 activation
Signaling by Interleukins
TNFs bind their physiological receptors
size
5
27
49
71
d
6C4 vs NI
PI5P, PP2A and IER3 Regulate PI3K/AKT Signaling
PI3K events in ERBB4 signaling
RAF/MAP kinase cascade
MAPK family signaling cascades
MAPK1/MAPK3 signaling
RAF-independent MAPK1/3 activation
Cytokine Signaling in Immune system
Class A/1 (Rhodopsin-like receptors)
GPCR ligand binding
Chemokine receptors bind chemokines
fold change
7.5
5.0
2.5
0.0
-2.5
category
Chemokine receptors bind chemokines
Class A/1 (Rhodopsin-like receptors)
Cytokine Signaling in Immune system
GPCR ligand binding
MAPK family signaling cascades
MAPK1/MAPK3 signaling
PI3K events in ERBB4 signaling
PI5P, PP2A and IER3 Regulate PI3K/AKT Signaling
RAF-independent MAPK1/3 activation
RAF/MAP kinase cascade
size
4
11
18
25

Supplementary Figure 4: Global transcription analysis of BMDMs infected with *M. tuberculosis* isolates 4I2 or 6C4. (A) Correlation heatmap generated from gene expression data for each of the non-infected BMDMs, BMDMs infected with *M. tuberculosis* isolate 4I2, and BMDMs infected with *M. tuberculosis* isolate 6C4 replicates. Pearson correlation coefficient is color coded and indicated on each cell. (B) Heatmap representing unsupervised hierarchical clustering of sample distribution and z-scores (adjusted p-value ≤0.05, up- (≥2) or down-regulated (≤-2) log fold). In total, 1366 genes were found to be significantly differentially regulated in one or both conditions: BMDMs infected with *M. tuberculosis* isolate 4I2 (dark red) or *M. tuberculosis* isolate 6C4 (blue) versus non-infected BMDMs (NI, yellow). Multiple testing adjustments were performed using the Benjamini-Hochberg (BH) procedure. (C,D) Plots representing the ten pathways most affected in BMDMs infected with *M. tuberculosis* isolates 4I2 (C) or 6C4 (D). Each pathway is represented by a beige node, connected to each gene associated with it by colored lines. Node size is associated with number of genes significantly expressed in the pathway. The legend indicates the color codes for each pathway/gene set. Fold change is used as a measure for significance in the altered genes, and it is color coded in each gene node. An MOI of 2 was used for infection.

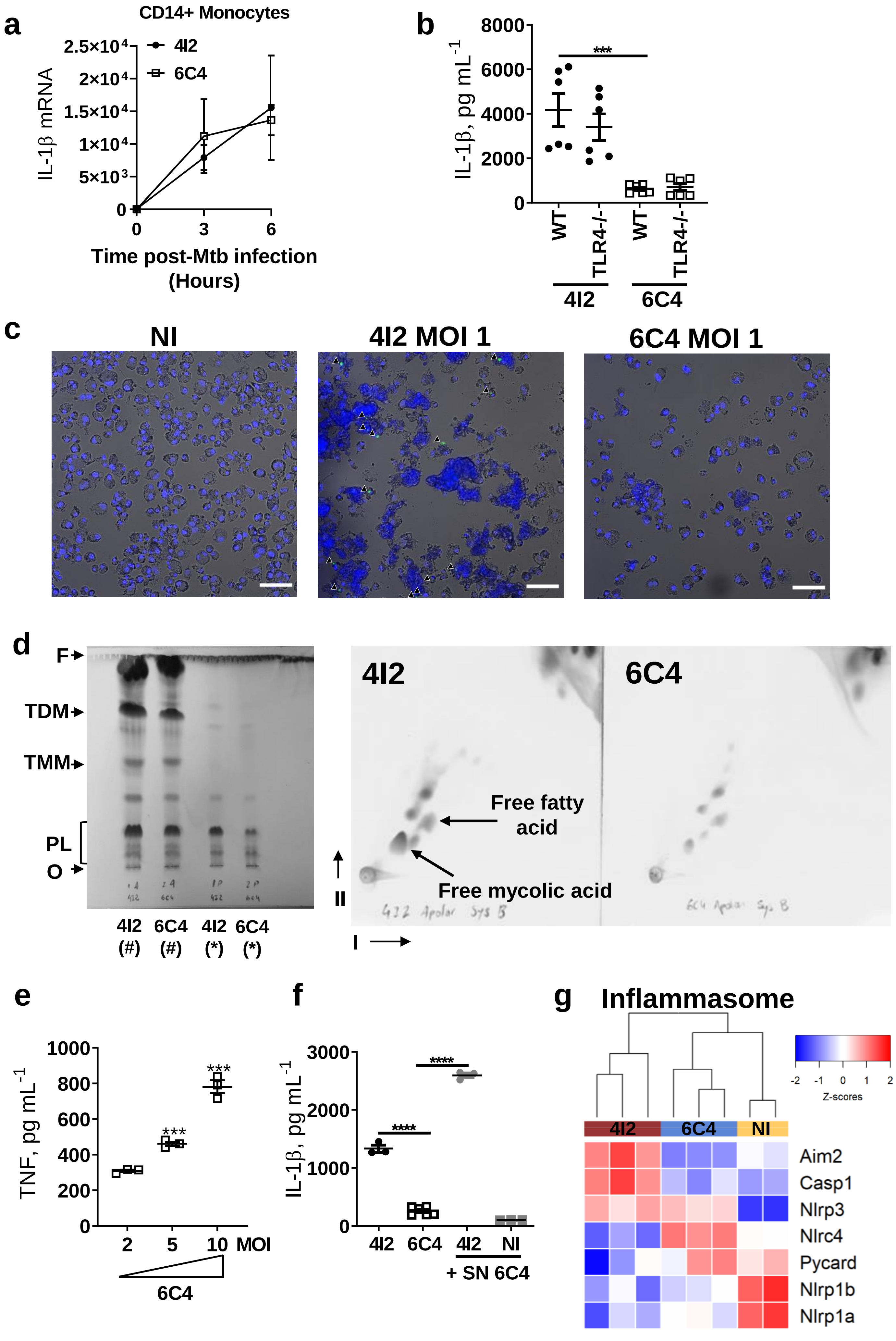
a
CD14+ Monocytes
IL-1β mRNA
2.5×10⁴
2×10⁴
1.5×10⁴
1×10⁴
5×10³
0
4I2
6C4
0
3
6
Time post-Mtb infection
(Hours)
b
IL-1β, pg mL-1
8000
6000
4000
2000
0
***
WT
TLR4-/-
WT
TLR4-/-
4I2
6C4
c
NI
4I2 MOI 1
6C4 MOI 1
d
F
TDM
TMM
PL
O
4I2
(#)
6C4
(#)
4I2
(*)
6C4
(*)
4I2
6C4
Free fatty
acid
Free mycolic acid
II
I
e
TNF, pg mL-1
1000
800
600
400
200
0
***
***
2
5
10 MOI
6C4
f
IL-1β, pg mL-1
3000
2000
1000
0
****
****
4I2
6C4
4I2
NI
+ SN 6C4
g
Inflammasome
-2
-1
0
1
2
Z-scores
4I2
6C4
NI
Aim2
Casp1
Nlrp3
Nlrc4
Pycard
Nlrp1b
Nlrp1a

Supplementary Figure 5: (A) CD14+ monocytes from PBMCs of 5 donors were infected with *M. tuberculosis* 4I2 (solid circles) or 6C4 (open squares) at a MOI of 1. At the indicated time points, RNA was extracted, converted to cDNA and subjected to real-time PCR. C57BL/6 WT (B, E, F, G) and TLR4 deficient (B) BMDMs were generated and infected with *M. tuberculosis* 4I2 or 6C4 as indicated. In (E) different MOI of bacteria were used, and in (F) the infection with *M. tuberculosis* 4I2 was combined with conditioned supernatant from BMDM cultures infected with *M. tuberculosis* 6C4 for 24h. (B,E,F) Twenty-four h post-infection the culture supernatants were harvested and the indicated cytokines measured by ELISA. (C) PMA-differentiated THP-1-ASC-GFP cells were infected with either *M. tuberculosis* isolate as indicated. Speck positive cells (white arrows) were identified and their percentage determined for the different conditions. Images are representative of two independent experiments. Scale bars correspond to 100 µm. (D) TLC of apolar (#) or polar (*) cell wall lipidic extracts obtained from *M. tuberculosis* 4I2 and 6C4 were run in a system consisting of chloroform/methanol/water (60:16:2) (Left). Free and apolar cell wall lipidic extracts were run in a 2D system B (direction 1: Petroleum ether 60-80/ acetone (92:8) – 3 runs; direction 2: Toluene/acetone (95:5) – 1 run) (Right). The experiment was performed twice with independent bacterial preparations and similar results. Annotated are TMM, trehalose monomycolate; TDM, trehalose dimycolate; PL, phospholipids; O, origin; F, solvent front; I, direction 1; II, direction 2. (G) Heatmap representing the expression of genes associated with the inflammasome pathway in non-infected BMDMs (NI; yellow) and BMDMs infected with *M. tuberculosis* 4I2 (dark red) or *M. tuberculosis* 6C4 (blue) for 6h. z-scores are color coded as shown. An MOI of 2 (A,B,F) or of 1 (C) was used for infection. Represented are the mean±SEM for different donors (A); for triplicate wells from 2 independent experiments (B) or from one experiment representative of two (E); and from one experiment (F). Statistical analysis was performed using two-tailed Student's *t*-test (*, $p<0.05$; **, $p<0.01$; ***, $p<0.001$; and ****, $p<0.0001$).

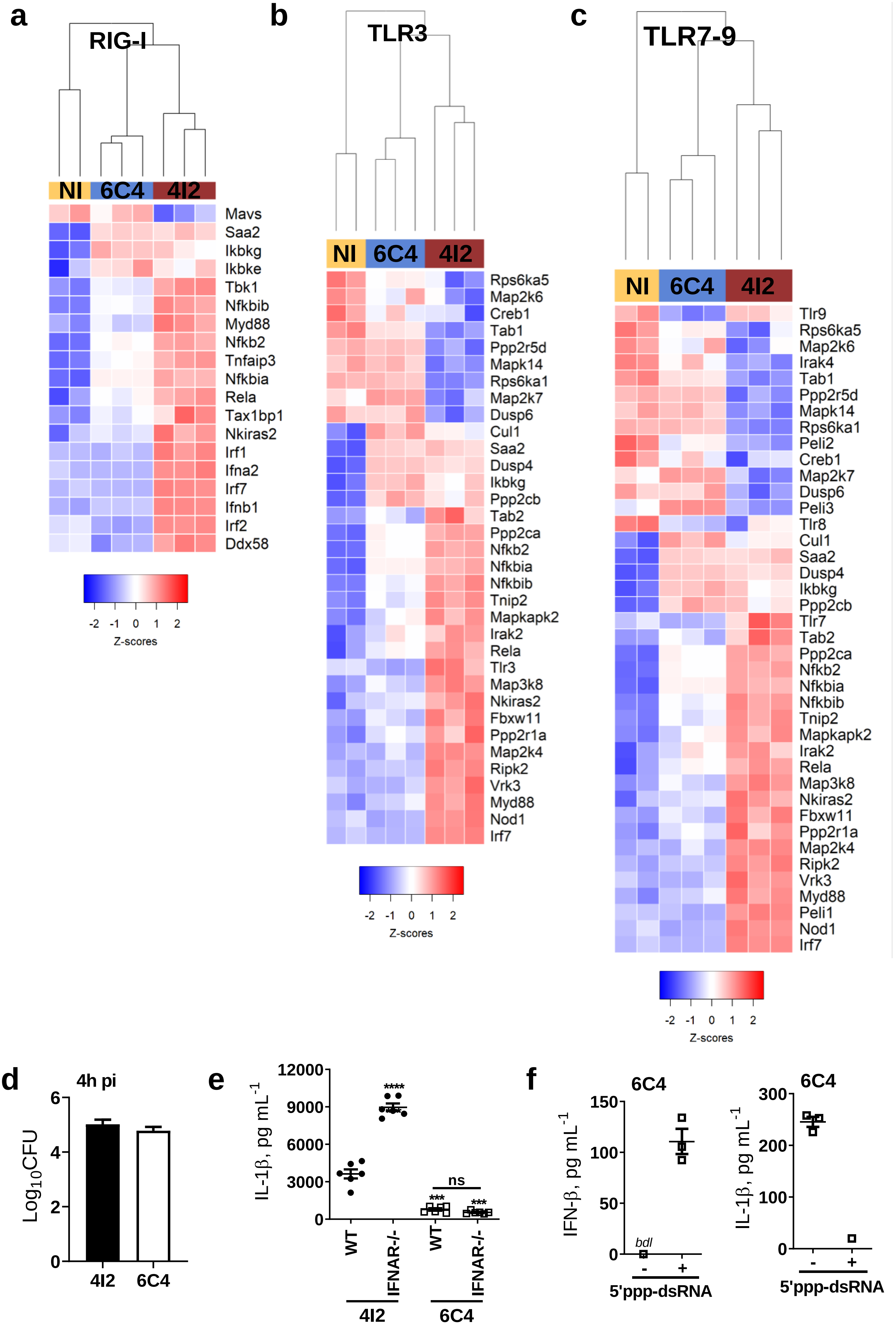
a
RIG-I
NI
6C4
4I2
Mavs
Saa2
Ikbkg
Ikbke
Tbk1
Nfkbib
Myd88
Nfkb2
Tnfaip3
Nfkbia
Rela
Tax1bp1
Nkiras2
Irf1
Ifna2
Irf7
Ifnb1
Irf2
Ddx58
-2 -1 0 1 2
Z-scores
b
TLR3
NI
6C4
4I2
Rps6ka5
Map2k6
Creb1
Tab1
Ppp2r5d
Mapk14
Rps6ka1
Map2k7
Dusp6
Cul1
Saa2
Dusp4
Ikbkg
Ppp2cb
Tab2
Ppp2ca
Nfkb2
Nfkbia
Nfkbib
Tnip2
Mapkapk2
Irak2
Rela
Tlr3
Map3k8
Nkiras2
Fbxw11
Ppp2r1a
Map2k4
Ripk2
Vrk3
Myd88
Nod1
Irf7
-2 -1 0 1 2
Z-scores
c
TLR7-9
NI
6C4
4I2
Tlr9
Rps6ka5
Map2k6
Irak4
Tab1
Ppp2r5d
Mapk14
Rps6ka1
Peli2
Creb1
Map2k7
Dusp6
Peli3
Tlr8
Cul1
Saa2
Dusp4
Ikbkg
Ppp2cb
Tlr7
Tab2
Ppp2ca
Nfkb2
Nfkbia
Nfkbib
Tnip2
Mapkapk2
Irak2
Rela
Map3k8
Nkiras2
Fbxw11
Ppp2r1a
Map2k4
Ripk2
Vrk3
Myd88
Peli1
Nod1
Irf7
-2 -1 0 1 2
Z-scores
d
4h pi
Log10CFU
0 2 4 6
4I2
6C4
e
IL-1β, pg mL-1
0 3000 6000 9000 12000
****
***
***
ns
WT
IFNAR-/-
WT
IFNAR-/-
4I2
6C4
f
6C4
IFN-β, pg mL-1
0 50 100 150
bdl
- +
5'ppp-dsRNA
6C4
IL-1β, pg mL-1
0 100 200 300
- +
5'ppp-dsRNA

Supplementary Figure 6. *M. tuberculosis* isolate 6C4 evades the macrophage cytosolic pathways. (A-C) 4I2 leads to an overall higher expression of genes in the RIG-I and TLR3/7/8/9 pathways. Heatmaps representing the expression of genes associated with the RIG-1 (A), TLR3 (B), TLR7, TLR8 and TLR9 (C) pathways in non-infected BMDMs (NI; yellow) and BMDMs infected with *M. tuberculosis* isolate 4I2 (dark red) or *M. tuberculosis* isolate 6C4 (blue) for 6h. z-scores are color coded as indicated in the legends. C57BL/6 WT (D,E,F) and IFNAR deficient (E) BMDMs were generated and infected with *M. tuberculosis* isolates 4I2 or 6C4 as indicated. (D) Four h post-infection, the cell cultures were extensively washed with PBS, the cells lysed and the intracellular bacteria quantified by CFU enumeration. (E,F) Twenty-four h post-infection the culture supernatants were harvested and the indicated cytokines measured by ELISA. An MOI of 2 was used for infections. In (F) the infection with *M. tuberculosis* isolate 6C4 was combined with 1µg/mL of the RIG-I agonist 5’ppp-dsRNA mixed with a transfection agent. Represented are the mean±SEM for triplicate wells from at least 2 independent experiments (D,E) or from 1 experiment (F). bdl, below detection level. Statistical analysis was performed using two-tailed Student’s t-test (*, $p<0.05$; **, $p<0.01$; ***, $p<0.001$; and ****, $p<0.0001$).

Supplementary Table 1: General characteristics and clinical features associated with selected *M. tuberculosis* isolates.

**MILD**										
**Chest X-ray**	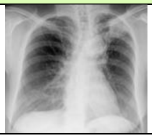	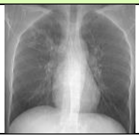	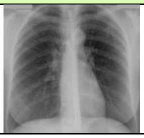	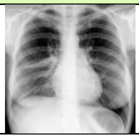	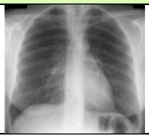	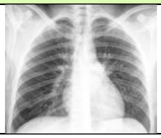	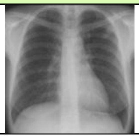	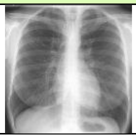	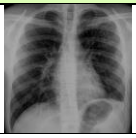	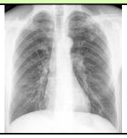
**Code**	**6D8**	**3A3**	**1I4**	**6D3**	**2I3**	**5A9**	**4I2**	**5F8**	**5D4**	**2D2**
**Sublineage**	L4/X	L4/LAM	L4/LAM	L4/LAM	L4/LAM	L4/LAM	L4/LAM	L4/LAM	L4/LAM	L4/LAM
**Gender**	Female	Female	Female	Female	Female	Male	Female	Female	Male	Male
**Age (years)**	44	35	39	30	39	25	34	26	28	45
**Time sympt. (w)**	10	2	10	24	12	5	1	8	26	13
**Clinical features**	Moderate lesions, low sympt., no anemia, low CRP	Minimal lesion, low sympt., no anemia, medium CRP	Minimal lesions, low sympt., no anemia, low CRP	Moderate lesions, low sympt., no anemia, low CRP	Minimal lesions, low sympt., no anemia, low CRP	Minimal lesions, low sympt., no anemia, low CRP	Moderate lesions, low sympt., no anemia, low CRP	Minimal lesions, low sympt., no anemia, low CRP	Minimal lesion, low sympt., no anemia, medium CRP	Minimal lesions, low sympt., no anemia, low CRP
**MODERATE**										
**Chest X-ray**	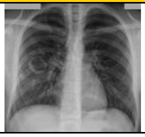	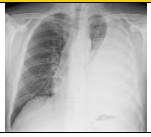	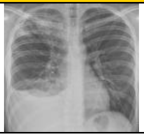	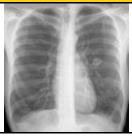	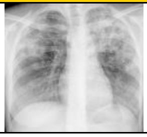	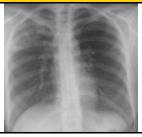	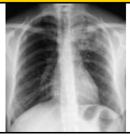	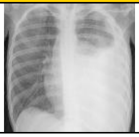	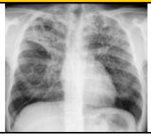	
**Code**	**5D3**	**1B2**	**5I5**	**3A8**	**1C7**	**3A7**	**5F9**	**1E1**	**2G8**	
**Sublineage**	L4/X	L4/LAM	L4/LAM	L4/LAM	L4/LAM	L4/LAM	L4/LAM	L4/LAM	L4/LAM	
**Gender**	Male	Male	Male	Female	Female	Female	Female	Female	Male	
**Age (years)**	22	35	22	31	20	28	50	27	25	
**Time sympt. (w)**	15	3	13	9	17	6	11	2	4	
**Clinical features**	Mod. lesions, low sympt., no anemia, medium CRP	Advanced lesions, no respir. failure, low sympt.	Moderate lesions, low sympt., no anemia, high CRP	Moderate lesions, low sympt., anemic, low CRP	Moderate lesions, low sympt., anemic, high CRP	Moderate lesions, low sympt., anemic, low CRP	Advanced lesions, no respir. failure, low sympt.	Advanced lesions, no respir. failure, low sympt.	Advanced lesions, no respir. failure, low sympt.	
**SEVERE**										
**Chest X-ray**	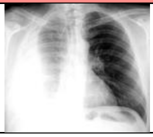	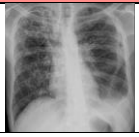	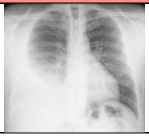	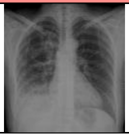	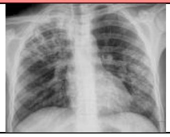	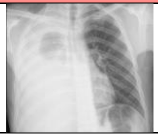	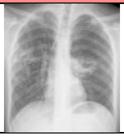			
**Code**	**5B5**	**5D6**	**5C8**	**5C7**	**6C1**	**6C4**	**4D5**			
**Sublineage**	L4/X	L4/other L4	L4/LAM	L4/LAM	L4/LAM	L4/LAM	L4/other L4			
**Gender**	Male	Male	Male	Female	Male	Male	Male			
**Age (years)**	54	43	42	49	19	20	19			
**Time sympt. (w)**	3	10	1	10	7	3	9			
**Clinical features**	Advanced lesions, no respir. failure, high sympt. / CRP	Advanced lesions, no respir. failure, high sympt. / CRP, anemic	Advanced lesions, no respir. failure, high sympt. / CRP	Advanced lesions, no respir. failure, high sympt. / CRP, anemic	Advanced lesions, no respir. failure, high sympt. / CRP, anemic	Advanced lesions, no respir. failure, high sympt. / CRP	Advanced lesions, no respir. failure, high sympt. / CRP			

Supplementary Table 2. List of genes with polymorpisms exclusively present in more than 30% of the low or high IL-1β inducing isolates

Genomic position	REF	ALT	Variant type	Gene name	Locus tag	Nucleotide substitution (HGVS)	Amino acid substitution	Protein product	Gene position	Gene length	Protein position	Protein length	Group_ID	Isolates with the polymorphism	Homoplasies	Creates a new TANNT box?
182849	A	C	missense variant	*fadE2*	*Rv0154c*	c.350T>G	p.L117R	acyl-CoA dehydrogenase FadE2	350	1212	117	403	High IL1B	1E1, 2G8, 5D4	No	No
618408	C	T	missense variant	*Rv0528*	*Rv0528*	c.104C>T	p.A35V	transmembrane protein	104	1590	35	529	Low IL1B	5D3, 6D8	No	No
619640	G	C	missense variant	*Rv0528*	*Rv0528*	c.1336G>C	p.V446L	transmembrane protein	1336	1590	446	529	Low IL1B	5I5, 2I3, 5A9	No	No
949486	C	T	missense variant	*pdc*	*Rv0853c*	c.1633G>A	p.E545K	alpha-keto-acid decarboxylase	1633	1683	545	560	Low IL1B	5C7	No	No
949535	C	T	synonymous variant	*pdc*	*Rv0853c*	c.1584G>A	p.A528A	alpha-keto-acid decarboxylase	1584	1683	528	560	Low IL1B	5D6	No	No
950230	C	T	missense variant	*pdc*	*Rv0853c*	c.889G>A	p.D297N	alpha-keto-acid decarboxylase	889	1683	297	560	Low IL1B	5B5, 5D3, 6D8	No	No
1256512	C	T	synonymous variant	*prpC*	*Rv1131*	c.381C>T	p.L127L	methylcitrate synthase PrpC	381	1182	127	393	Low IL1B	5F8	No	Yes
1256806	C	T	synonymous variant	*prpC*	*Rv1131*	c.675C>T	p.D225D	methylcitrate synthase PrpC	675	1182	225	393	Low IL1B	5B5, 5D3, 6D8	No	No
1257078	T	C	missense variant	*prpC*	*Rv1131*	c.947T>C	p.V316A	methylcitrate synthase PrpC	947	1182	316	393	Low IL1B	5C7	No	No
2360894	G	C	missense variant	*helZ*	*Rv2101*	c.655G>C	p.G219R	helicase HelZ	655	3042	219	1013	Low IL1B	1C7	No	No
2361604	G	C	synonymous variant	*helZ*	*Rv2101*	c.1365G>C	p.V455V	helicase HelZ	1365	3042	455	1013	Low IL1B	5D6	No	No
2361648	A	C	missense variant	*helZ*	*Rv2101*	c.1409A>C	p.E470A	helicase HelZ	1409	3042	470	1013	Low IL1B	1C7, 3A7, 5F9, 6C4	No	No
2362041	A	C	missense variant	*helZ*	*Rv2101*	c.1802A>C	p.Q601P	helicase HelZ	1802	3042	601	1013	Low IL1B	5D6	No	No
2415656	C	G	missense variant	*murD*	*Rv2155c*	c.739G>C	p.G247R	UDP-N-acetylmuramoylalanine--D-glutamate ligase	739	1461	247	486	Low IL1B	5D6	No	No
2415797	C	T	missense variant	*murD*	*Rv2155c*	c.598G>A	p.A200T	UDP-N-acetylmuramoylalanine--D-glutamate ligase	598	1461	200	486	Low IL1B	5I5, 2I3, 5A9	No	No
2416156	G	A	missense variant	*murD*	*Rv2155c*	c.239C>T	p.T80I	UDP-N-acetylmuramoylalanine--D-glutamate ligase	239	1461	80	486	Low IL1B	5B5, 5D3, 6D8	Yes (2)	No
2582348	G	T	intergenic region	*metV-Rv2309A*	*Rvnt24-Rv2309A*	n.2582348G>T	--	intergenic region	--	--	--	--	Low IL1B	5B5, 5D3, 6D8	No	No
2582695	C	A	intergenic region	*metV-Rv2309A*	*Rvnt24-Rv2309A*	n.2582695C>A	--	intergenic region	--	--	--	--	Low IL1B	5I5, 2I3, 5A9	No	No
2585127	AC	A	frameshift variant	*Rv2313c*	*Rv2313c*	c.779delG	p.G260fs	hypothetical protein	779	855	260	284	Low IL1B	5I5, 2I3, 5A9	No	
2585668	A	C	missense variant	*Rv2313c*	*Rv2313c*	c.239T>G	p.L80R	hypothetical protein	239	855	80	284	Low IL1B	3A7, 5F9, 6C4	No	No
2722175	G	A	synonymous variant	*Rv2425c*	*Rv2425c*	c.1134C>T	p.A378A	hypothetical protein	1134	1443	378	480	Low IL1B	5C7	Yes (3)	Yes
2723229	G	A	missense variant	*Rv2425c*	*Rv2425c*	c.80C>T	p.A27V	hypothetical protein	80	1443	27	480	Low IL1B	1C7, 3A7, 5F9, 6C4	No	No
2760152	G	A	missense variant	*mmuM*	*Rv2458*	c.374G>A	p.C125Y	homocysteine S-methyltransferase MmuM	374	909	125	302	Low IL1B	5D6	No	No
2760241	G	A	missense variant	*mmuM*	*Rv2458*	c.463G>A	p.E155K	homocysteine S-methyltransferase MmuM	463	909	155	302	Low IL1B	5B5, 5D3, 6D8	No	No
2760273	G	T	synonymous variant	*mmuM*	*Rv2458*	c.495G>T	p.A165A	homocysteine S-methyltransferase MmuM	495	909	165	302	Low IL1B	5B5	Yes (2)	No
2760642	T	C	synonymous variant	*mmuM*	*Rv2458*	c.864T>C	p.I288I	homocysteine S-methyltransferase MmuM	864	909	288	302	Low IL1B	1C7	No	No
2773673	T	G	missense variant	*aglA*	*Rv2471*	c.110T>G	p.F37C	alpha-glucosidase AglA	110	1641	37	546	Low IL1B	5D3, 6D8	No	No
2773818	A	C	synonymous variant	*aglA*	*Rv2471*	c.255A>C	p.A85A	alpha-glucosidase AglA	255	1641	85	546	Low IL1B	5I5, 2I3, 5A9	No	No
2773931	T	C	missense variant	*aglA*	*Rv2471*	c.368T>C	p.M123T	alpha-glucosidase AglA	368	1641	123	546	Low IL1B	5B5	No	No
2775042	G	A	synonymous variant	*aglA*	*Rv2471*	c.1479G>A	p.A493A	alpha-glucosidase AglA	1479	1641	493	546	Low IL1B	5C7	No	No
2793187	G	A	stop gained	*lipQ*	*Rv2485c*	c.802C>T	p.Q268*	carboxylesterase LipQ	802	1266	268	421	Low IL1B	5D3, 6D8	No	No
2793473	G	C	missense variant	*lipQ*	*Rv2485c*	c.516C>G	p.I172M	carboxylesterase LipQ	516	1266	172	421	Low IL1B	5I5, 2I3, 5A9	No	No
2885349	G	A	missense variant	*Rv2565*	*Rv2565*	c.739G>A	p.V247M	NTE family protein	739	1752	247	583	Low IL1B	3A7, 5F9, 6C4	No	No
2885734	G	A	missense variant	*Rv2565*	*Rv2565*	c.1124G>A	p.G375D	NTE family protein	1124	1752	375	583	Low IL1B	5B5, 5D3, 6D8	No	No
2890127	G	A	synonymous variant	*Rv2567*	*Rv2567*	c.333G>A	p.L111L	hypothetical protein	333	2655	111	884	Low IL1B	1C7	No	No
2891177	C	T	synonymous variant	*Rv2567*	*Rv2567*	c.1383C>T	p.S461S	hypothetical protein	1383	2655	461	884	Low IL1B	3A7, 5F9, 6C4	Yes (2)	No
2891267	T	C	synonymous variant	*Rv2567*	*Rv2567*	c.1473T>C	p.G491G	hypothetical protein	1473	2655	491	884	Low IL1B	5D6	No	No
2891728	G	A	missense variant	*Rv2567*	*Rv2567*	c.1934G>A	p.R645Q	hypothetical protein	1934	2655	645	884	Low IL1B	5D6	No	No
3017071	G	A	missense variant	*ppgK*	*Rv2702*	c.214G>A	p.G72S	polyphosphate glucokinase	214	798	72	265	Low IL1B	3A8	No	No
3017276	A	G	missense variant	*ppgK*	*Rv2702*	c.419A>G	p.N140S	polyphosphate glucokinase	419	798	140	265	Low IL1B	5B5, 5D3, 6D8	No	No
3017465	C	T	missense variant	*ppgK*	*Rv2702*	c.608C>T	p.T203I	polyphosphate glucokinase	608	798	203	265	Low IL1B	5D6	No	No
3105748	C	T	missense variant	*Rv2797c*	*Rv2797c*	c.1560G>A	p.M520I	hypothetical protein	1560	1689	520	562	Low IL1B	5B5, 5D3, 6D8	No	No
3106789	G	T	synonymous variant	*Rv2797c*	*Rv2797c*	c.519C>A	p.G173G	hypothetical protein	519	1689	173	562	Low IL1B	1C7, 3A7, 5F9, 6C4	No	No
3197427	C	G	missense variant	*amiC*	*Rv2888c*	c.859G>C	p.E287Q	amidase AmiC	859	1422	287	473	Low IL1B	3A7, 5F9, 6C4	No	No
3198059	C	T	missense variant	*amiC*	*Rv2888c*	c.227G>A	p.G76E	amidase AmiC	227	1422	76	473	Low IL1B	5D3, 6D8	No	No
3461873	A	G	missense variant	*Rv3093c*	*Rv3093c*	c.892T>C	p.C298R	oxidoreductase	892	1005	298	334	Low IL1B	5I5, 5A9	No	No
3462135	C	G	missense variant	*Rv3093c*	*Rv3093c*	c.630G>C	p.W210C	oxidoreductase	630	1005	210	334	Low IL1B	5D6	No	No
3462145	A	AGGCGC	frameshift variant	*Rv3093c*	*Rv3093c*	c.615 619dupGCGCC	p.L207fs	oxidoreductase	619	1005	207	334	Low IL1B	5B5, 6D8	No	
3601898	C	A	missense variant	*Rv3225c*	*Rv3225c*	c.543G>T	p.E181D	GCN5-like N-acetyltransferase	543	1425	181	474	High IL1B	1E1, 2G8, 2D2, 5D4	No	No
3887774	G	A	synonymous variant	*ilvB2*	*Rv3470c*	c.1029C>T	p.R343R	acetolactate synthase large subunit	1029	1659	343	552	Low IL1B	1C7	No	No
3887921	A	G	synonymous variant	*ilvB2*	*Rv3470c*	c.882T>C	p.F294F	acetolactate synthase large subunit	882	1659	294	552	Low IL1B	5B5, 5D3, 6D8	No	No
3887975	G	A	synonymous variant	*ilvB2*	*Rv3470c*	c.828C>T	p.C276C	acetolactate synthase large subunit	828	1659	276	552	Low IL1B	5D6	No	No
3939526	G	A	intergenic region	*Rv3510c-PE PGRS55*	*Rv3510c-Rv3511*	n.3939526G>A	--	intergenic region	--	--	--	--	High IL1B	1E1, 2G8, 2D2, 5D4	No	No
3939590	T	G	intergenic region	*Rv3510c-PE PGRS55*	*Rv3510c-Rv3511*	n.3939590T>G	--	intergenic region	--	--	--	--	High IL1B	5D4	No	No
3966248	G	A	missense variant	*Rv3529c*	*Rv3529c*	c.791C>T	p.S264L	hypothetical protein	791	1155	264	384	Low IL1B	5F8	No	No
3966303	A	C	missense variant	*Rv3529c*	*Rv3529c*	c.736T>G	p.Y246D	hypothetical protein	736	1155	246	384	Low IL1B	3A7, 5F9, 6C4	No	No
3966513	G	A	stop gained	*Rv3529c*	*Rv3529c*	c.526C>T	p.Q176*	hypothetical protein	526	1155	176	384	Low IL1B	5I5, 2I3, 5A9	No	No
3972735	G	A	synonymous variant	*hsaF*	*Rv3534c*	c.858C>T	p.S286S	4-hydroxy-2-oxovalerate aldolase	858	1041	286	346	Low IL1B	3A7, 5F9, 6C4	Yes (5)	Yes
3973492	A	G	missense variant	*hsaF*	*Rv3534c*	c.101T>C	p.V34A	4-hydroxy-2-oxovalerate aldolase	101	1041	34	346	Low IL1B	5D3, 6D8	No	No
4102233	A	C	synonymous variant	*dppD*	*Rv3663c*	c.1446T>G	p.G482G	dipeptide ABC transporter ATP-binding protein DppD	1446	1647	482	548	Low IL1B	5D3, 6D8	No	No
4102246	TGCAGATCGGCCA	T	disruptive inframe deletion	*dppD*	*Rv3663c*	c.1421 1432delTGGCCGATCTGC	p.L474 L477del	dipeptide ABC transporter ATP-binding protein DppD	1432	1647	474	548	Low IL1B	1C7	No	
4102792	C	A	missense variant	*dppD*	*Rv3663c*	c.887G>T	p.G296V	dipeptide ABC transporter ATP-binding protein DppD	887	1647	296	548	Low IL1B	5B5	No	No
4103228	G	A	missense variant	*dppD*	*Rv3663c*	c.451C>T	p.P151S	dipeptide ABC transporter ATP-binding protein DppD	451	1647	151	548	Low IL1B	3A7, 5F9, 6C4	No	No

Supplementary Table 3. List of polymorphisms in genes of the ESX-I secretion system and exclusively associated with high or low IL-1β-inducing M. tuberculosis isolates.

Genomic position	REF	ALT	Variant type	Gene name	Locus tag	Nucleotide substitution (HGVS)	Amino acid substitution	Protein product	Gene position	Gene length	Protein position	Protein length	Group_ID	Isolates with the polymorphism
2068715	C	T	synonymous variant	*secA2*	*Rv1821*	c.2259C>T	p.I753I	accessory Sec system translocase SecA2	2259	2427	753	808	Low IL1B	1C7
2170803	C	T	intergenic region	*PPE35-Rv1919c*	*Rv1918c-Rv1919c*	n.2170803C>T	--	--	--	--	--	--	Low IL1B	3A7, 5F9, 6C4
4055801	A	G	missense variant	*espA*	*Rv3616c*	c.575T>C	p.I192T	ESX-1 secretion-associated protein EspA	575	1179	192	392	Low IL1B	5D6
4323355	G	C	intergenic region	*Rv3848-espR*	*Rv3848-Rv3849*	n.4323355G>C	--	--	--	--	--	--	Low IL1B	5B5, 5D3, 6D8
4323931	G	A	intergenic region	*espR-Rv3850*	*Rv3849-Rv3850*	n.4323931G>A	--	--	--	--	--	--	Low IL1B	3A7, 5F9, 6C4
4340149	G	A	intergenic region	*Rv3863-espE*	*Rv3863-Rv3864*	n.4340149G>A	--	--	--	--	--	--	High IL1B	4D5
4356110	C	G	synonymous variant	*eccD1*	*Rv3877*	c.1104C>G	p.L368L	ESX-1 secretion system protein EccD1	1104	1536	368	511	Low IL1B	5D6
4357123	C	A	missense variant	*espJ*	*Rv3878*	c.431C>A	p.T144K	ESX-1 secretion-associated protein EspJ	431	843	144	280	Low IL1B	5B5, 5D3, 6D8

Supplementary Table 4. List of polymorphisms (SNPs + indels) identified between *M. tuberculosis* isolates 4I2 and 6C4.

Genomic position	REF	ALT	Variant type	Gene name	Locus tag	Nucleotide substitution (HGVS)	Amino acid substitution	Protein product	Gene position	Gene length	Protein position	Protein length	Group_ID	Strain_ID
6817	G	A	synonymous variant	*gyrB*	*Rv0005*	c.1578G>A	p.K526K	DNA gyrase subunit B	1578	2028	526	675	High IL1B	4I2
29588	A	G	missense variant	*Rv0025*	*Rv0025*	c.344A>G	p.Y115C	hypothetical protein	344	363	115	120	High IL1B	4I2
30944	C	T	missense variant	*Rv0026*	*Rv0026*	c.1223C>T	p.P408L	hypothetical protein	1223	1347	408	448	High IL1B	4I2
38213	G	T	missense variant	*fadD34*	*Rv0035*	c.955G>T	p.G319C	fatty-acid--CoA ligase FadD34	955	1689	319	562	High IL1B	4I2
40162	C	T	missense variant	*Rv0037c*	*Rv0037c*	c.1041G>A	p.M347I	MFS-type transporter	1041	1326	347	441	High IL1B	4I2
49478	CCGTTG	C	frameshift variant	*Rv0045c*	*Rv0045c*	c.457_461delCAACG	p.Q153fs	hydrolase	461	897	153	298	High IL1B	4I2
90502	C	T	missense variant	*Rv0083*	*Rv0083*	c.103C>T	p.H35Y	oxidoreductase	103	1923	35	640	High IL1B	4I2
128671	G	A	missense variant	*ctpI*	*Rv0107c*	c.1871C>T	p.A624V	cation-transporter ATPase I	1871	4899	624	1632	High IL1B	4I2
135542	G	A	synonymous variant	*Rv0111*	*Rv0111*	c.1593G>A	p.S531S	acyltransferase	1593	2058	531	685	High IL1B	4I2
168877	T	C	missense variant	*Rv0143c*	*Rv0143c*	c.1306A>G	p.M436V	transmembrane protein	1306	1479	436	492	High IL1B	4I2
174253	C	T	missense variant	*Rv0147*	*Rv0147*	c.1016C>T	p.A339V	aldehyde dehydrogenase	1016	1521	339	506	High IL1B	4I2
228847	G	A	missense variant	*Rv0194*	*Rv0194*	c.1970G>A	p.R657H	multidrug ABC transporter ATPase/permease	1970	3585	657	1194	High IL1B	4I2
253388	C	G	missense variant	*pckA*	*Rv0211*	c.1607C>G	p.A536G	phosphoenolpyruvate carboxykinase	1607	1821	536	606	High IL1B	4I2
282188	G	A	missense variant	*Rv0235c*	*Rv0235c*	c.427C>T	p.R143C	transmembrane protein	427	1449	143	482	High IL1B	4I2
290121	C	A	synonymous variant	*htdX*	*Rv0241c*	c.534G>T	p.P178P	3-hydroxyacyl-thioester dehydratase HtdX	534	843	178	280	High IL1B	4I2
299587	G	A	missense variant	*Rv0248c*	*Rv0248c*	c.1217C>T	p.P406L	succinate dehydrogenase flavoprotein subunit	1217	1941	406	646	High IL1B	4I2
332131	C	A	stop gained	*Rv0276*	*Rv0276*	c.384C>A	p.Y128*	hypothetical protein	384	921	128	306	High IL1B	4I2
347766	C	T	missense variant	*eccC3*	*Rv0284*	c.2132C>T	p.A711V	ESX-3 secretion system protein EccC3	2132	3993	711	1330	High IL1B	4I2
365358	G	A	missense variant	*Rv0303*	*Rv0303*	c.125G>A	p.G42D	dehydrogenase/reductase	125	909	42	302	High IL1B	4I2
411118	CCGCGG	C	frameshift variant	*iniA*	*Rv0342*	c.286_290delGCGCG	p.A96fs	isoniazid inductible protein IniA	286	1923	96	640	High IL1B	4I2
435726	C	T	synonymous variant	*purA*	*Rv0357c*	c.1044G>A	p.E348E	adenylosuccinate synthetase	1044	1299	348	432	High IL1B	4I2
468189	G	A	intergenic region	*Rv0386-purT*	*Rv0386-Rv0389*	n.468189G>A	--	--	--	--	--	--	High IL1B	4I2
583956	G	C	synonymous variant	*Rv0493c*	*Rv0493c*	c.735C>G	p.G245G	hypothetical protein	735	990	245	329	High IL1B	4I2
632330	G	T	synonymous variant	*Rv0539*	*Rv0539*	c.588G>T	p.R196R	dolichyl-phosphate sugar synthase	588	633	196	210	High IL1B	4I2
637128	G	T	synonymous variant	*pitA*	*Rv0545c*	c.336C>A	p.G112G	low-affinity inorganic phosphate transporter	336	1254	112	417	High IL1B	4I2
660859	C	T	synonymous variant	*cyp135B1*	*Rv0568*	c.1410C>T	p.G470G	cytochrome P450 Cyp135B1	1410	1419	470	472	High IL1B	4I2
663907	G	A	synonymous variant	*Rv0571c*	*Rv0571c*	c.912C>T	p.D304D	hypothetical protein	912	1332	304	443	High IL1B	4I2
669395	C	T	synonymous variant	*Rv0575c*	*Rv0575c*	c.351G>A	p.V117V	oxidoreductase	351	1167	117	388	High IL1B	4I2
757080	A	G	missense variant	*atsD*	*Rv0663*	c.944A>G	p.Y315C	arylsulfatase AtsD	944	2364	315	787	High IL1B	4I2
767480	C	G	intergenic region	*rpoC-Rv0669c*	*Rv0668-Rv0669c*	n.767480C>G	--	--	--	--	--	--	High IL1B	4I2
784440	G	T	synonymous variant	*fusA1*	*Rv0684*	c.1956G>T	p.A652A	elongation factor G	1956	2106	652	701	High IL1B	4I2
842847	T	C	missense variant	*mmsB*	*Rv0751c*	c.385A>G	p.T129A	3-hydroxyisobutyrate dehydrogenase	385	885	129	294	High IL1B	4I2
853579	C	G	missense variant	*phoR*	*Rv0758*	c.1184C>G	p.A395G	two component system response sensor kinase PhoR	1184	1458	395	485	High IL1B	4I2
859383	C	T	missense variant	*cyp123*	*Rv0766c*	c.690G>A	p.M230I	cytochrome P450 Cyp123	690	1209	230	402	High IL1B	4I2
922044	G	A	synonymous variant	*Rv0830*	*Rv0830*	c.75G>A	p.Q25Q	S-adenosylmethionine-dependent methyltransferase	75	906	25	301	High IL1B	4I2
922933	CA	C	frameshift variant	*Rv0831c*	*Rv0831c*	c.776delT	p.M259fs	hypothetical protein	776	816	259	271	High IL1B	4I2
990533	T	C	synonymous variant	*Rv0890c*	*Rv0890c*	c.2064A>G	p.T688T	HTH-type transcriptional regulator	2064	2649	688	882	High IL1B	4I2
1000086	C	T	synonymous variant	*gltA2*	*Rv0896*	c.615C>T	p.F205F	citrate synthase 1	615	1296	205	431	High IL1B	4I2
1041129	C	T	intergenic region	*pstS2-pstB*	*Rv0932c-Rv0933*	n.1041129C>T	--	--	--	--	--	--	High IL1B	4I2
1055049	C	T	missense variant	*pgi*	*Rv0946c*	c.1637G>A	p.R546H	glucose-6-phosphate isomerase	1637	1662	546	553	High IL1B	4I2
1070832	G	A	missense variant	*Rv0958*	*Rv0958*	c.950G>A	p.R317Q	magnesium chelatase	950	1380	317	459	High IL1B	4I2
1076020	C	A	missense variant	*Rv0963c*	*Rv0963c*	c.78G>T	p.K26N	hypothetical protein	78	801	26	266	High IL1B	4I2
1076658	G	GC	frameshift variant	*Rv0964c*	*Rv0964c*	c.20dupG	p.G8fs	hypothetical protein	20	483	7	160	High IL1B	4I2
1084476	C	T	synonymous variant	*accA2*	*Rv0973c*	c.1275G>A	p.L425L	acetyl/propionyl-CoA carboxylase subuit alpha	1275	2004	425	667	High IL1B	4I2
1102674	G	A	missense variant	*Rv0987*	*Rv0987*	c.133G>A	p.D45N	adhesion component ABC transporter permease	133	2568	45	855	High IL1B	4I2
1112530	T	C	synonymous variant	*Rv0996*	*Rv0996*	c.147T>C	p.A49A	transmembrane protein	147	1077	49	358	High IL1B	4I2
1132368	C	T	synonymous variant	*pks16*	*Rv1013*	c.744C>T	p.T248T	polyketide synthase	744	1635	248	544	High IL1B	4I2
1243693	C	T	intergenic region	*Rv1120c-zwf1*	*Rv1120c-Rv1121*	n.1243693C>T	--	--	--	--	--	--	High IL1B	4I2
1332623	G	A	missense variant	*sigI*	*Rv1189*	c.532G>A	p.V178I	ECF RNA polymerase sigma factor SigI	532	873	178	290	High IL1B	4I2
1336882	C	T	synonymous variant	*fadD36*	*Rv1193*	c.1089C>T	p.D363D	fatty-acid--CoA ligase FadD36	1089	1422	363	473	High IL1B	4I2
1338700	A	G	intergenic region	*Rv1194c-PE13*	*Rv1194c-Rv1195*	n.1338700A>G	--	--	--	--	--	--	High IL1B	4I2
1373170	G	C	missense variant	*Rv1230c*	*Rv1230c*	c.1028C>G	p.P343R	membrane protein	1028	1236	343	411	High IL1B	4I2
1374936	CA	C	frameshift variant	*Rv1232c*	*Rv1232c*	c.1232delT	p.L411fs	hypothetical protein	1232	1308	411	435	High IL1B	4I2
1423805	G	C	synonymous variant	*Rv1273c*	*Rv1273c*	c.246C>G	p.T82T	drug ABC transporter ATP-binding protein	246	1749	82	582	High IL1B	4I2
1447774	C	T	synonymous variant	*argS*	*Rv1292*	c.1396C>T	p.L466L	arginine--tRNA ligase	1396	1653	466	550	High IL1B	4I2
1513845	T	G	missense variant	*irtA*	*Rv1348*	c.799T>G	p.S267A	iron ABC transporter ATP-binding protein/permease IrtA	799	2580	267	859	High IL1B	4I2
1536681	C	T	missense variant	*Rv1364c*	*Rv1364c*	c.964G>A	p.A322T	sigma factor regulatory protein	964	1962	322	653	High IL1B	4I2
1558564	G	T	synonymous variant	*carB*	*Rv1384*	c.1464G>T	p.L488L	carbamoyl-phosphate synthase large subunit	1464	3348	488	1115	High IL1B	4I2
1595877	G	A	synonymous variant	*uvrC*	*Rv1420*	c.1836G>A	p.K612K	excinuclease ABC subunit UvrC	1836	1941	612	646	High IL1B	4I2
1605108	C	G	synonymous variant	*Rv1429*	*Rv1429*	c.231C>G	p.A77A	hypothetical protein	231	1269	77	422	High IL1B	4I2
1625347	C	CG	frameshift variant	*opcA*	*Rv1446c*	c.18dupC	p.D7fs	OXPP cycle protein OpcA	18	912	6	303	High IL1B	4I2
1724120	G	A	synonymous variant	*pks5*	*Rv1527c*	c.4290C>T	p.D1430D	polyketide synthase	4290	6327	1430	2108	High IL1B	4I2
1755599	C	T	missense variant	*plsB1*	*Rv1551*	c.155C>T	p.A52V	acyltransferase PlsB	155	1866	52	621	High IL1B	4I2
1767373	A	G	missense variant	*treY*	*Rv1563c*	c.2060T>C	p.F687S	maltooligosyl trehalose synthase	2060	2298	687	765	High IL1B	4I2
1769099	C	A	missense variant	*treY*	*Rv1563c*	c.334G>T	p.D112Y	maltooligosyl trehalose synthase	334	2298	112	765	High IL1B	4I2
1771320	G	A	synonymous variant	*treX*	*Rv1564c*	c.282C>T	p.D94D	maltooligosyl trehalose synthase	282	2166	94	721	High IL1B	4I2
1824946	G	T	synonymous variant	*cydA*	*Rv1623c*	c.942C>A	p.I314I	cytochrome D ubiquinol oxidase subunit I CydA	942	1458	314	485	High IL1B	4I2
1964111	C	T	missense variant	*narX*	*Rv1736c*	c.76G>A	p.E26K	nitrate reductase-like protein NarX	76	1959	26	652	High IL1B	4I2
2013079	T	C	synonymous variant	*Rv1779c*	*Rv1779c*	c.1401A>G	p.P467P	integral membrane protein	1401	1794	467	597	High IL1B	4I2
2019942	A	G	missense variant	*eccC5*	*Rv1783*	c.686A>G	p.Q229R	ESX-5 type VII secretion system protein EccC5	686	4176	229	1391	High IL1B	4I2
2058257	T	G	start lost	*Rv1816*	*Rv1816*	c.2T>G	p.L1?	HTH-type transcriptional regulator	2	705	1	234	High IL1B	4I2
2084526	G	A	synonymous variant	*Rv1836c*	*Rv1836c*	c.111C>T	p.P37P	hypothetical protein	111	2034	37	677	High IL1B	4I2
2140763	T	C	synonymous variant	*Rv1894c*	*Rv1894c*	c.1107A>G	p.A369A	hypothetical protein	1107	1131	369	376	High IL1B	4I2
2151838	G	C	missense variant	*aao*	*Rv1905c*	c.558C>G	p.D186E	D-amino acid oxidase	558	963	186	320	High IL1B	4I2
2231879	T	C	missense variant	*erm(37)*	*Rv1988*	c.200T>C	p.V67A	23S rRNA (adenine(2058)-N(6))-methyltransferase Erm(37)	200	540	67	179	High IL1B	4I2
2241807	C	CGGT	disruptive inframe insertion	*ctpF*	*Rv1997*	c.1652_1654dupTGG	p.V551dup	cation transporter ATPase F	1655	2718	552	905	High IL1B	4I2
2243744	T	G	intergenic region	*Rv1998c-Rv1999c*	*Rv1998c-Rv1999c*	n.2243744T>G	--	--	--	--	--	--	High IL1B	4I2
2246298	A	G	missense variant	*Rv2000*	*Rv2000*	c.1090A>G	p.R364G	hypothetical protein	1090	1614	364	537	High IL1B	4I2
2278442	C	G	synonymous variant	*Rv2030c*	*Rv2030c*	c.45G>C	p.R15R	hypothetical protein	45	2046	15	681	High IL1B	4I2
2280584	G	A	missense variant	*Rv2033c*	*Rv2033c*	c.499C>T	p.H167Y	hypothetical protein	499	843	167	280	High IL1B	4I2
2318079	T	C	missense variant	*cobN*	*Rv2062c*	c.2675A>G	p.E892G	cobalamin biosynthesis protein CobN	2675	3585	892	1194	High IL1B	4I2
2320344	G	A	missense variant	*cobN*	*Rv2062c*	c.410C>T	p.T137I	cobalamin biosynthesis protein CobN	410	3585	137	1194	High IL1B	4I2
2327492	C	T	stop retained variant	*cobK*	*Rv2070c*	c.734G>A	p.*245*	precorrin-6A reductase	734	735	245	244	High IL1B	4I2
2327492	C	T	synonymous variant	*sigC*	*Rv2069*	c.549C>T	p.L183L	ECF RNA polymerase sigma factor SigC	549	558	183	185	High IL1B	4I2
2332384	G	A	missense variant	*Rv2075c*	*Rv2075c*	c.496C>T	p.R166C	hypothetical protein	496	1464	166	487	High IL1B	4I2
2337027	G	A	missense variant	*Rv2079*	*Rv2079*	c.1673G>A	p.R558H	hypothetical protein	1673	1971	558	656	High IL1B	4I2
2385695	C	T	missense variant	*metH*	*Rv2124c*	c.373G>A	p.G125R	methionine synthase	373	3579	125	1192	High IL1B	4I2
2411367	C	T	synonymous variant	*murC*	*Rv2152c*	c.756G>A	p.P252P	UDP-N-acetylmuramate--alanine ligase	756	1485	252	494	High IL1B	4I2
2420535	C	A	missense variant	*murE*	*Rv2158c*	c.74G>T	p.G25V	UDP-N-acetylmuramoylalanyl-D-glutamate--2,6-diaminopimelate ligase	74	1608	25	535	High IL1B	4I2
2421395	T	C	missense variant	*Rv2159c*	*Rv2159c*	c.271A>G	p.M91V	hypothetical protein	271	1035	91	344	High IL1B	4I2
2434023	T	C	missense variant	*Rv2172c*	*Rv2172c*	c.514A>G	p.M172V	hypothetical protein	514	906	172	301	High IL1B	4I2
2443188	G	A	synonymous variant	*Rv2180c*	*Rv2180c*	c.27C>T	p.H9H	integral membrane protein	27	888	9	295	High IL1B	4I2

2465611	G	A	synonymous variant	*asnB*	*Rv2201*	c.615G>A	p.Q205Q	asparagine synthetase	615	1959	205	652	High IL1B	4I2
2468058	C	T	intergenic region	*adoK-Rv2203*	*Rv2202c-Rv2203*	n.2468058C>T	--	--	--	--	--	--	High IL1B	4I2
2470886	AAACTTAGCCGCG	A	disruptive inframe deletion	*Rv2206*	*Rv2206*	c.267_278delACTTAGCCGCGA	p.L90_E93del	transmembrane protein	267	711	89	236	High IL1B	4I2
2483744	A	G	missense variant	*Rv2216*	*Rv2216*	c.119A>G	p.N40S	epimerase family protein	119	906	40	301	High IL1B	4I2
2490095	T	C	synonymous variant	*glnE*	*Rv2221c*	c.2259A>G	p.T753T	[glutamate--ammonia-ligase] adenylyltransferase	2259	2985	753	994	High IL1B	4I2
2562756	A	T	missense variant	*lppO*	*Rv2290*	c.158A>T	p.E53V	lipoprotein LppO	158	516	53	171	High IL1B	4I2
2587568	C	T	missense variant	*Rv2315c*	*Rv2315c*	c.1237G>A	p.D413N	hypothetical protein	1237	1518	413	505	High IL1B	4I2
2595291	G	A	intergenic region	*rocE-Rv2323c*	*Rv2320c-Rv2323c*	n.2595291G>A	--	--	--	--	--	--	High IL1B	4I2
2649353	A	G	missense variant	*phoH1*	*Rv2368c*	c.622T>C	p.Y208H	phosphate starvation-inducible protein PhoH	622	1059	208	352	High IL1B	4I2
2653724	G	A	synonymous variant	*dnaJ2*	*Rv2373c*	c.264C>T	p.G88G	chaperone protein DnaJ	264	1149	88	382	High IL1B	4I2
2662183	C	G	missense variant	*mbtE*	*Rv2380c*	c.4933G>C	p.A1645P	peptide synthetase	4933	5049	1645	1682	High IL1B	4I2
2747680	G	A	missense variant	*valS*	*Rv2448c*	c.2546C>T	p.A849V	valine--tRNA ligase	2546	2631	849	876	High IL1B	4I2
2811167	G	A	synonymous variant	*bkdA*	*Rv2497c*	c.930C>T	p.L310L	3-methyl-2-oxobutanoate dehydrogenase subunit alpha	930	1104	310	367	High IL1B	4I2
2823610	G	A	synonymous variant	*Rv2508c*	*Rv2508c*	c.984C>T	p.A328A	integral membrane protein	984	1338	328	445	High IL1B	4I2
2825268	C	G	synonymous variant	*Rv2509*	*Rv2509*	c.591C>G	p.A197A	short-chain type dehydrogenase/reductase	591	807	197	268	High IL1B	4I2
2841549	G	T	missense variant	*fas*	*Rv2524c*	c.7784C>A	p.T2595N	fatty acid synthase	7784	9210	2595	3069	High IL1B	4I2
2851937	G	A	synonymous variant	*mrr*	*Rv2528c*	c.735C>T	p.G245G	restriction system protein	735	921	245	306	High IL1B	4I2
2925824	G	A	missense variant	*vapC40*	*Rv2596*	c.91G>A	p.V31I	ribonuclease VapC40	91	405	31	134	High IL1B	4I2
2980970	C	A	missense variant	*Rv2660c*	*Rv2660c*	c.221G>T	p.C74F	hypothetical protein	221	228	74	75	High IL1B	4I2
2992863	C	T	missense variant	*hemY*	*Rv2677c*	c.1130G>A	p.G377D	protoporphyrinogen oxidase	1130	1359	377	452	High IL1B	4I2
3059225	T	C	missense variant	*pgsA3*	*Rv2746c*	c.7A>G	p.R3G	CDP-diacylglycerol--glycerol-3-phosphate 3-phosphatidyltransferase	7	630	3	209	High IL1B	4I2
3089679	G	A	missense variant	*pepR*	*Rv2782c*	c.683C>T	p.P228L	zinc protease	683	1317	228	438	High IL1B	4I2
3119081	T	G	intergenic region	*Rv2813-Rv2816c*	*Rv2813-Rv2816c*	n.3119081T>G	--	--	--	--	--	--	High IL1B	4I2
3158935	G	C	missense variant	*Rv2850c*	*Rv2850c*	c.1120C>G	p.R374G	magnesium chelatase	1120	1890	374	629	High IL1B	4I2
3176744	G	A	synonymous variant	*Rv2864c*	*Rv2864c*	c.522C>T	p.A174A	penicillin-binding lipoprotein	522	1812	174	603	High IL1B	4I2
3214392	A	G	intergenic region	*rplS-lppW*	*Rv2904c-Rv2905*	n.3214392A>G	--	--	--	--	--	--	High IL1B	4I2
3267743	A	G	missense variant	*ppsE*	*Rv2935*	c.7A>G	p.I3V	phthiocerol synthesis polyketide synthase type I PpsE	7	4467	3	1488	High IL1B	4I2
3270533	T	G	missense variant	*ppsE*	*Rv2935*	c.2797T>G	p.S933A	phthiocerol synthesis polyketide synthase type I PpsE	2797	4467	933	1488	High IL1B	4I2
3275269	G	C	synonymous variant	*papA5*	*Rv2939*	c.321G>C	p.L107L	phthiocerol/phthiodiolone dimycocerosyl transferase	321	1269	107	422	High IL1B	4I2
3285551	T	C	missense variant	*mmpL7*	*Rv2942*	c.482T>C	p.M161T	transmembrane transport protein MmpL7	482	2763	161	920	High IL1B	4I2
3294302	G	A	synonymous variant	*pks1*	*Rv2946c*	c.2052C>T	p.S684S	polyketide synthase	2052	4851	684	1616	High IL1B	4I2
3302324	AGCGAG	A	frameshift variant	*fadD29*	*Rv2950c*	c.127_131delCTCGC	p.L43fs	long-chain-fatty-acid--AMP ligase FadD29	131	1860	43	619	High IL1B	4I2
3317366	G	T	missense variant	*purU*	*Rv2964*	c.838G>T	p.G280C	formyltetrahydrofolate deformylase	838	933	280	310	High IL1B	4I2
3374859	G	A	missense variant	*Rv3015c*	*Rv3015c*	c.806C>T	p.P269L	hypothetical protein	806	1014	269	337	High IL1B	4I2
3498640	G	A	missense variant	*devS*	*Rv3132c*	c.626C>T	p.A209V	two component sensor histidine kinase DevS	626	1737	209	578	High IL1B	4I2
3503246	A	G	missense variant	*Rv3136A*	*Rv3136A*	c.32T>C	p.L11P	hypothetical protein	32	333	11	110	High IL1B	4I2
3504930	C	T	synonymous variant	*pflA*	*Rv3138*	c.736C>T	p.L246L	pyruvate formate lyase activating protein PflA	736	1089	246	362	High IL1B	4I2
3514512	G	C	missense variant	*nuoD*	*Rv3148*	c.1175G>C	p.G392A	NADH-quinone oxidoreductase subunit D	1175	1323	392	440	High IL1B	4I2
3519294	G	GGCC	disruptive inframe insertion	*nuoH*	*Rv3152*	c.14_16dupGCC	p.G5_H6insR	NADH-quinone oxidoreductase subunit H	17	1233	6	410	High IL1B	4I2
3526678	G	A	missense variant	*nuoN*	*Rv3158*	c.889G>A	p.A297T	NADH-quinone oxidoreductase subunit N	889	1596	297	531	High IL1B	4I2
3554787	G	A	missense variant	*Rv3189*	*Rv3189*	c.146G>A	p.G49E	hypothetical protein	146	621	49	206	High IL1B	4I2
3556885	A	G	missense variant	*Rv3190A*	*Rv3190A*	c.31A>G	p.K11E	hypothetical protein	31	210	11	69	High IL1B	4I2
3560035	G	A	intergenic region	*Rv3192-Rv3193c*	*Rv3192-Rv3193c*	n.3560035G>A	--	--	--	--	--	--	High IL1B	4I2
3561155	G	A	missense variant	*Rv3193c*	*Rv3193c*	c.2018C>T	p.A673V	transmembrane protein	2018	2979	673	992	High IL1B	4I2
3604533	T	C	missense variant	*aroA*	*Rv3227*	c.1157T>C	p.M386T	3-phosphoshikimate 1-carboxyvinyltransferase	1157	1353	386	450	High IL1B	4I2
3619842	G	A	synonymous variant	*secA1*	*Rv3240c*	c.690C>T	p.G230G	protein translocase subunit SecA	690	2850	230	949	High IL1B	4I2
3661530	C	T	synonymous variant	*birA*	*Rv3279c*	c.483G>A	p.Q161Q	bifunctional biotin operon repressor/biotin--[acetyl-CoA-carboxylase] synthetase	483	801	161	266	High IL1B	4I2
3705806	A	C	missense variant	*sdhA*	*Rv3318*	c.807A>C	p.E269D	succinate dehydrogenase flavoprotein subunit	807	1773	269	590	High IL1B	4I2
3710250	G	A	intergenic region	*moaC3-Rv3327*	*Rv3324c-Rv3327*	n.3710250G>A	--	--	--	--	--	--	High IL1B	4I2
3794607	T	A	synonymous variant	*dxs2*	*Rv3379c*	c.261A>T	p.A87A	1-deoxy-D-xylulose-5-phosphate synthase	261	1611	87	536	High IL1B	4I2
3798126	G	A	missense variant	*idsB*	*Rv3383c*	c.364C>T	p.P122S	polyprenyl synthetase IdsB	364	1053	122	350	High IL1B	4I2
3829152	G	A	synonymous variant	*guaB3*	*Rv3410c*	c.759C>T	p.L253L	oxidoreductase	759	1128	253	375	High IL1B	4I2
3901479	C	T	missense variant	*Rv3482c*	*Rv3482c*	c.628G>A	p.E210K	membrane protein	628	783	210	260	High IL1B	4I2
3964463	AG	A	intergenic region	*Rv3527-Rv3528c*	*Rv3527-Rv3528c*	n.3964464delG	--	--	--	--	--	--	High IL1B	4I2
4012954	G	A	missense variant	*kshB*	*Rv3571*	c.538G>A	p.A180T	3-ketosteroid-9-alpha-hydroxylase reductase subunit	538	1077	180	358	High IL1B	4I2
4043365	G	T	synonymous variant	*Rv3600c*	*Rv3600c*	c.495C>A	p.A165A	type III pantothenate kinase	495	819	165	272	High IL1B	4I2
4090452	G	T	missense variant	*Rv3649*	*Rv3649*	c.1672G>T	p.A558S	helicase	1672	2316	558	771	High IL1B	4I2
4152943	T	C	missense variant	*ask*	*Rv3709c*	c.541A>G	p.S181G	aspartokinase	541	1266	181	421	High IL1B	4I2
4174816	C	T	intergenic region	*Rv3727-Rv3728*	*Rv3727-Rv3728*	n.4174816C>T	--	--	--	--	--	--	High IL1B	4I2
4218350	T	C	missense variant	*Rv3773c*	*Rv3773c*	c.476A>G	p.K159R	hypothetical protein	476	585	159	194	High IL1B	4I2
4235631	C	A	synonymous variant	*Rv3789*	*Rv3789*	c.258C>A	p.L86L	GtrA family protein	258	366	86	121	High IL1B	4I2
4239763	C	T	missense variant	*aftA*	*Rv3792*	c.1832C>T	p.T611M	arabinofuranosyltransferase	1832	1932	611	643	High IL1B	4I2
4252095	G	T	synonymous variant	*fadE35*	*Rv3797*	c.1011G>T	p.A337A	acyl-CoA dehydrogenase FadE35	1011	1782	337	593	High IL1B	4I2
4287370	A	G	missense variant	*Rv3822*	*Rv3822*	c.650A>G	p.Q217R	hypothetical protein	650	1215	217	404	High IL1B	4I2
4288405	G	T	missense variant	*mmpL8*	*Rv3823c*	c.3125C>A	p.A1042E	integral membrane transport protein MmpL8	3125	3270	1042	1089	High IL1B	4I2
4359195	G	A	synonymous variant	*espK*	*Rv3879c*	c.588C>T	p.G196G	ESX-1 secretion-associated protein EspK	588	2190	196	729	High IL1B	4I2
4372558	G	A	missense variant	*Rv3888c*	*Rv3888c*	c.149C>T	p.T50I	membrane protein	149	1026	50	341	High IL1B	4I2
4373475	C	G	synonymous variant	*espG2*	*Rv3889c*	c.156G>C	p.V52V	ESX-2 secretion-associated protein EspG2	156	831	52	276	High IL1B	4I2
4377447	G	A	synonymous variant	*eccC2*	*Rv3894c*	c.3006C>T	p.D1002D	ESX-2 type VII secretion system protein EccC	3006	4191	1002	1396	High IL1B	4I2
4377865	A	C	missense variant	*eccC2*	*Rv3894c*	c.2588T>G	p.I863S	ESX-2 type VII secretion system protein EccC	2588	4191	863	1396	High IL1B	4I2
4385177	G	C	missense variant	*Rv3899c*	*Rv3899c*	c.203C>G	p.P68R	hypothetical protein	203	1233	68	410	High IL1B	4I2
4403900	A	G	missense variant	*cwlM*	*Rv3915*	c.709A>G	p.M237V	peptidoglycan hydrolase	709	1221	237	406	High IL1B	4I2
4410200	G	A	synonymous variant	*Rv3922c*	*Rv3922c*	c.216C>T	p.L72L	membrane protein insertion efficiency factor	216	363	72	120	High IL1B	4I2
10628	A	G	missense variant	*Rv0007*	*Rv0007*	c.715A>G	p.N239D	membrane protein	715	915	239	304	Low IL1B	6C4
27352	C	T	missense variant	*whiB5*	*Rv0022c*	c.91G>A	p.E31K	transcriptional regulator WhiB5	91	420	31	139	Low IL1B	6C4
55533	T	TGCC	conservative inframe insertion	*ponA1*	*Rv0050*	c.1888_1890dupCCG	p.P630dup	bifunctional penicillin-insensitive transglycosylase/penicillin-sensitive transpeptidase	1891	2037	631	678	Low IL1B	6C4
107183	C	T	synonymous variant	*Rv0097*	*Rv0097*	c.450C>T	p.T150T	oxidoreductase	450	870	150	289	Low IL1B	6C4
163320	A	G	intergenic region	*Rv0135c-cyp138*	*Rv0135c-Rv0136*	n.163320A>G	--	--	--	--	--	--	Low IL1B	6C4
170263	G	A	intergenic region	*Rv0143c-Rv0144*	*Rv0143c-Rv0144*	n.170263G>A	--	--	--	--	--	--	Low IL1B	6C4
183401	C	G	intergenic region	*fadE2-pntAa*	*Rv0154c-Rv0155*	n.183401C>G	--	--	--	--	--	--	Low IL1B	6C4
197047	A	G	missense variant	*yrbE1A*	*Rv0167*	c.187A>G	p.M63V	membrane protein	187	798	63	265	Low IL1B	6C4
247872	C	T	missense variant	*Rv0207c*	*Rv0207c*	c.241G>A	p.A81T	hypothetical protein	241	729	81	242	Low IL1B	6C4
251019	G	A	synonymous variant	*Rv0210*	*Rv0210*	c.900G>A	p.L300L	hypothetical protein	900	1479	300	492	Low IL1B	6C4
255703	A	C	missense variant	*Rv0213c*	*Rv0213c*	c.248T>G	p.L83R	methyltransferase	248	1314	83	437	Low IL1B	6C4
269001	C	T	synonymous variant	*Rv0225*	*Rv0225*	c.339C>T	p.G113G	hypothetical protein	339	1155	113	384	Low IL1B	6C4
349411	G	A	synonymous variant	*eccC3*	*Rv0284*	c.3777G>A	p.L1259L	ESX-3 secretion system protein EccC3	3777	3993	1259	1330	Low IL1B	6C4
393208	G	A	missense variant	*cyp135A1*	*Rv0327c*	c.838C>T	p.H280Y	cytochrome P450 Cyp135A1	838	1350	280	449	Low IL1B	6C4
393780	T	G	missense variant	*cyp135A1*	*Rv0327c*	c.266A>C	p.E89A	cytochrome P450 Cyp135A1	266	1350	89	449	Low IL1B	6C4
396100	G	T	intergenic region	*Rv0330c-Rv0331*	*Rv0330c-Rv0331*	n.396100G>T	--	--	--	--	--	--	Low IL1B	6C4
500870	A	G	missense variant	*thiE*	*Rv0414c*	c.149T>C	p.L50P	thiamine-phosphate synthase	149	669	50	222	Low IL1B	6C4
535045	C	G	missense variant	*Rv0446c*	*Rv0446c*	c.171G>C	p.W57C	transmembrane protein	171	771	57	256	Low IL1B	6C4
572887	C	G	missense variant	*lprQ*	*Rv0483*	c.1178C>G	p.A393G	lipoprotein LprQ	1178	1356	393	451	Low IL1B	6C4
599162	A	G	missense variant	*mmpL2*	*Rv0507*	c.1964A>G	p.Q655R	transmembrane transport protein MmpL2	1964	2907	655	968	Low IL1B	6C4
638214	C	G	missense variant	*Rv0547c*	*Rv0547c*	c.703G>C	p.A235P	oxidoreductase	703	885	235	294	Low IL1B	6C4
650012	C	T	synonymous variant	*menH*	*Rv0558*	c.324C>T	p.D108D	demethylmenaquinone methyltransferase	324	705	108	234	Low IL1B	6C4

651475	G	A	synonymous variant	*Rv0560c*	*Rv0560c*	c.30C>T	p.A10A	benzoquinone methyltransferase		30	726	10	241	Low IL1B	6C4
683140	G	A	missense variant	*Rv0585c*	*Rv0585c*	c.1133C>T	p.A378V	integral membrane protein		1133	2388	378	795	Low IL1B	6C4
691887	G	GCC	frameshift variant	*mce2D*	*Rv0592*	c.1393 1394dupCC	p.P466fs	Mce family protein Mce2D		1395	1527	465	508	Low IL1B	6C4
770573	C	T	intergenic region	*end-lpqP*	*Rv0670-Rv0671*	n.770573C>T	--	--	--	--	--	--		Low IL1B	6C4
780469	C	T	intergenic region	*Rv0680c-Rv0681*	*Rv0680c-Rv0681*	n.780469C>T	--	--	--	--	--	--		Low IL1B	6C4
815889	C	T	missense variant	*sppA*	*Rv0724*	c.227C>T	p.T76M	protease IV SppA		227	1872	76	623	Low IL1B	6C4
816379	C	T	synonymous variant	*sppA*	*Rv0724*	c.717C>T	p.G239G	protease IV SppA		717	1872	239	623	Low IL1B	6C4
831206	C	T	stop gained	*Rv0739*	*Rv0739*	c.352C>T	p.Q118*	hypothetical protein		352	807	118	268	Low IL1B	6C4
858085	G	A	synonymous variant	*Rv0765c*	*Rv0765c*	c.780C>T	p.P260P	oxidoreductase		780	828	260	275	Low IL1B	6C4
1002044	A	G	synonymous variant	*Rv0897c*	*Rv0897c*	c.372T>C	p.D124D	oxidoreductase		372	1608	124	535	Low IL1B	6C4
1088262	C	T	missense variant	*fadE13*	*Rv0975c*	c.235G>A	p.A79T	acyl-CoA dehydrogenase FadE13		235	1149	79	382	Low IL1B	6C4
1106649	T	C	missense variant	*grcC2*	*Rv0989c*	c.734A>G	p.Y245C	polyprenyl-diphosphate synthase GrcC		734	978	245	325	Low IL1B	6C4
1150143	G	T	missense variant	*kdpD*	*Rv1028c*	c.1544C>A	p.A515D	sensor protein KdpD		1544	2583	515	860	Low IL1B	6C4
1184755	G	A	synonymous variant	*Rv1061*	*Rv1061*	c.741G>A	p.L247L	hypothetical protein		741	864	247	287	Low IL1B	6C4
1198740	G	C	missense variant	*fadA3*	*Rv1074c*	c.634C>G	p.R212G	beta-ketoacyl CoA thiolase FadA		634	1218	212	405	Low IL1B	6C4
1207765	C	T	missense variant	*Rv1084*	*Rv1084*	c.130C>T	p.R44C	hypothetical protein		130	2022	44	673	Low IL1B	6C4
1238654	C	G	missense variant	*Rv1112*	*Rv1112*	c.400C>G	p.L134V	GTP-binding protein		400	1074	134	357	Low IL1B	6C4
1274335	G	A	synonymous variant	*Rv1146*	*Rv1146*	c.981G>A	p.L327L	transmembrane transport protein		981	1413	327	470	Low IL1B	6C4
1288401	C	A	missense variant	*narG*	*Rv1161*	c.1074C>A	p.D358E	nitrate reductase subunit alpha		1074	3699	358	1232	Low IL1B	6C4
1364434	C	T	missense variant	*sigE*	*Rv1221*	c.22C>T	p.R8W	ECF RNA polymerase sigma factor SigE		22	774	8	257	Low IL1B	6C4
1374438	G	A	missense variant	*Rv1231c*	*Rv1231c*	c.427C>T	p.R143C	membrane protein		427	543	143	180	Low IL1B	6C4
1404817	C	A	missense variant	*Rv1257c*	*Rv1257c*	c.1268G>T	p.W423L	oxidoreductase		1268	1368	423	455	Low IL1B	6C4
1423687	C	T	missense variant	*Rv1273c*	*Rv1273c*	c.364G>A	p.V122I	drug ABC transporter ATP-binding protein		364	1749	122	582	Low IL1B	6C4
1439711	G	A	missense variant	*Rv1286*	*Rv1286*	c.805G>A	p.A269T	adenylyl-sulfate kinase		805	1845	269	614	Low IL1B	6C4
1455097	A	C	intergenic region	*rho-rpmE*	*Rv1297-Rv1298*	n.1455097A>C	--	--	--	--	--	--		Low IL1B	6C4
1525069	G	T	intergenic region	*Rv1356c-Rv1357c*	*Rv1356c-Rv1357c*	n.1525069G>T	--	--	--	--	--	--		Low IL1B	6C4
1533836	A	G	intergenic region	*PPE19-Rv1362c*	*Rv1361c-Rv1362c*	n.1533836A>G	--	--	--	--	--	--		Low IL1B	6C4
1612081	T	C	synonymous variant	*Rv1433*	*Rv1433*	c.648T>C	p.Y216Y	hypothetical protein		648	816	216	271	Low IL1B	6C4
1692824	A	G	intergenic region	*Rv1501-Rv1502*	*Rv1501-Rv1502*	n.1692824A>G	--	--	--	--	--	--		Low IL1B	6C4
1735849	C	T	intergenic region	*Rv1534-Rv1535*	*Rv1534-Rv1535*	n.1735849C>T	--	--	--	--	--	--		Low IL1B	6C4
1756626	T	C	synonymous variant	*plsB1*	*Rv1551*	c.1182T>C	p.S394S	acyltransferase PlsB		1182	1866	394	621	Low IL1B	6C4
1766351	A	G	synonymous variant	*treZ*	*Rv1562c*	c.792T>C	p.L264L	malto-oligosyltrehalose trehalohydrolase		792	1743	264	580	Low IL1B	6C4
1860092	G	C	missense variant	*pheT*	*Rv1650*	c.335G>C	p.S112T	phenylalanine--tRNA ligase subunit beta		335	2496	112	831	Low IL1B	6C4
1906266	C	T	intergenic region	*moeX-Rv1682*	*Rv1681-Rv1682*	n.1906266C>T	--	--	--	--	--	--		Low IL1B	6C4
1953648	T	C	missense variant	*Rv1727*	*Rv1727*	c.379T>C	p.W127R	hypothetical protein		379	570	127	189	Low IL1B	6C4
1972021	G	A	intergenic region	*idi-pknF*	*Rv1745c-Rv1746*	n.1972021G>A	--	--	--	--	--	--		Low IL1B	6C4
2121902	C	A	intergenic region	*Rv1871c-lldD2*	*Rv1871c-Rv1872c*	n.2121902C>A	--	--	--	--	--	--		Low IL1B	6C4
2122395	C	T	missense variant	*lldD2*	*Rv1872c*	c.757G>A	p.V253M	L-lactate dehydrogenase		757	1245	253	414	Low IL1B	6C4
2123110	T	C	synonymous variant	*lldD2*	*Rv1872c*	c.42A>G	p.A14A	L-lactate dehydrogenase		42	1245	14	414	Low IL1B	6C4
2133668	C	A	missense variant	*Rv1883c*	*Rv1883c*	c.25G>T	p.G9C	hypothetical protein		25	462	9	153	Low IL1B	6C4
2148391	C	A	missense variant	*cinA*	*Rv1901*	c.730C>A	p.P244T	competence damage-inducible protein CinA		730	1293	244	430	Low IL1B	6C4
2170803	C	T	intergenic region	*PPE35-Rv1919c*	*Rv1918c-Rv1919c*	n.2170803C>T	--	--	--	--	--	--		Low IL1B	6C4
2175725	G	A	missense variant	*lipD*	*Rv1923*	c.553G>A	p.G185R	lipase LipD		553	1341	185	446	Low IL1B	6C4
2180125	G	A	synonymous variant	*Rv1927*	*Rv1927*	c.453G>A	p.K151K	hypothetical protein		453	774	151	257	Low IL1B	6C4
2189438	A	G	missense variant	*Rv1937*	*Rv1937*	c.943A>G	p.T315A	oxygenase		943	2520	315	839	Low IL1B	6C4
2199684	G	A	intergenic region	*Rv1948c-Rv1950c*	*Rv1948c-Rv1950c*	n.2199684G>A	--	--	--	--	--	--		Low IL1B	6C4
2210740	A	C	missense variant	*mce3B*	*Rv1967*	c.140A>C	p.N47T	Mce family protein Mce3B		140	1029	47	342	Low IL1B	6C4
2222944	G	A	missense variant	*Rv1979c*	*Rv1979c*	c.221C>T	p.T74I	permease		221	1446	74	481	Low IL1B	6C4
2223123	C	T	synonymous variant	*Rv1979c*	*Rv1979c*	c.42G>A	p.L14L	permease		42	1446	14	481	Low IL1B	6C4
2236364	T	C	missense variant	*ctpG*	*Rv1992c*	c.943A>G	p.I315V	cation transporter ATPase G		943	2316	315	771	Low IL1B	6C4
2316912	G	A	synonymous variant	*Rv2061c*	*Rv2061c*	c.174C>T	p.N58N	hypothetical protein		174	405	58	134	Low IL1B	6C4
2361648	A	C	missense variant	*helZ*	*Rv2101*	c.1409A>C	p.E470A	helicase HelZ		1409	3042	470	1013	Low IL1B	6C4
2432567	C	G	synonymous variant	*Rv2170*	*Rv2170*	c.333C>G	p.G111G	GCN5-like N-acetyltransferase		333	621	111	206	Low IL1B	6C4
2437712	G	T	synonymous variant	*Rv2175c*	*Rv2175c*	c.175C>A	p.R59R	DNA-binding protein		175	441	59	146	Low IL1B	6C4
2452290	G	A	missense variant	*Rv2190c*	*Rv2190c*	c.983C>T	p.P328L	endopeptidase		983	1158	328	385	Low IL1B	6C4
2454611	GT	G	frameshift variant	*Rv2191*	*Rv2191*	c.794delT	p.V265fs	hypothetical protein		794	1938	265	645	Low IL1B	6C4
2585668	A	C	missense variant	*Rv2313c*	*Rv2313c*	c.239T>G	p.L80R	hypothetical protein		239	855	80	284	Low IL1B	6C4
2616140	G	C	missense variant	*mmpL9*	*Rv2339*	c.1448G>C	p.G483A	transmembrane transport protein MmpL9		1448	2889	483	962	Low IL1B	6C4
2723229	G	A	missense variant	*Rv2425c*	*Rv2425c*	c.80C>T	p.A27V	hypothetical protein		80	1443	27	480	Low IL1B	6C4
2806259	A	G	intergenic region	*PE_PGRS43-Rv2490a*	*Rv2490c-Rv2490a*	n.2806259A>G	--	--	--	--	--	--		Low IL1B	6C4
2821152	G	A	missense variant	*fadD35*	*Rv2505c*	c.445C>T	p.P149S	fatty-acid--CoA ligase FadD35		445	1644	149	547	Low IL1B	6C4
2851965	G	A	missense variant	*mrr*	*Rv2528c*	c.707C>T	p.A236V	restriction system protein		707	921	236	306	Low IL1B	6C4
2885349	G	A	missense variant	*Rv2565*	*Rv2565*	c.739G>A	p.V247M	NTE family protein		739	1752	247	583	Low IL1B	6C4
2891177	C	T	synonymous variant	*Rv2567*	*Rv2567*	c.1383C>T	p.S461S	hypothetical protein		1383	2655	461	884	Low IL1B	6C4
2931803	T	C	missense variant	*snoP*	*Rv2604c*	c.487A>G	p.I163V	glutamine amidotransferase SnoP		487	597	163	198	Low IL1B	6C4
3002123	C	G	stop gained	*arsB1*	*Rv2685*	c.141C>G	p.Y47*	arsenic-transport integral membrane protein ArsB		141	1287	47	428	Low IL1B	6C4
3025657	T	C	missense variant	*sthA*	*Rv2713*	c.217T>C	p.Y73H	pyridine nucleotide transhydrogenase		217	1407	73	468	Low IL1B	6C4
3044835	G	A	missense variant	*Rv2732c*	*Rv2732c*	c.155C>T	p.P52L	transmembrane protein		155	615	52	204	Low IL1B	6C4
3063653	G	T	missense variant	*Rv2751*	*Rv2751*	c.16G>T	p.A6S	hypothetical protein		16	891	6	296	Low IL1B	6C4
3106789	G	T	synonymous variant	*Rv2797c*	*Rv2797c*	c.519C>A	p.G173G	hypothetical protein		519	1689	173	562	Low IL1B	6C4
3118976	C	G	missense variant	*Rv2813*	*Rv2813*	c.753C>G	p.D251E	hypothetical protein		753	813	251	270	Low IL1B	6C4
3189497	C	G	intergenic region	*mpt53-cdsA*	*Rv2878c-Rv2881c*	n.3189497C>G	--	--	--	--	--	--		Low IL1B	6C4
3197427	C	G	missense variant	*amiC*	*Rv2888c*	c.859G>C	p.E287Q	amidase AmiC		859	1422	287	473	Low IL1B	6C4
3198877	G	A	synonymous variant	*tsf*	*Rv2889c*	c.231C>T	p.D77D	elongation factor EF-Ts		231	816	77	271	Low IL1B	6C4
3216661	G	A	synonymous variant	*rimM*	*Rv2907c*	c.231C>T	p.D77D	16S rRNA processing protein RimM		231	531	77	176	Low IL1B	6C4
3237024	C	G	synonymous variant	*smc*	*Rv2922c*	c.783G>C	p.R261R	chromosome partition protein Smc		783	3618	261	1205	Low IL1B	6C4
3244674	G	A	synonymous variant	*fadD26*	*Rv2930*	c.978G>A	p.R326R	fatty-acid--CoA ligase FadD26		978	1752	326	583	Low IL1B	6C4
3263997	C	G	missense variant	*ppsD*	*Rv2934*	c.1750C>G	p.L584V	phthiocerol synthesis polyketide synthase type I PpsD		1750	5484	584	1827	Low IL1B	6C4
3268930	G	C	missense variant	*ppsE*	*Rv2935*	c.1194G>C	p.L398F	phthiocerol synthesis polyketide synthase type I PpsE		1194	4467	398	1488	Low IL1B	6C4
3281814	A	G	missense variant	*mas*	*Rv2940c*	c.902T>C	p.V301A	multifunctional mycocerosic acid synthase		902	6336	301	2111	Low IL1B	6C4
3285945	C	G	synonymous variant	*mmpL7*	*Rv2942*	c.876C>G	p.A292A	transmembrane transport protein MmpL7		876	2763	292	920	Low IL1B	6C4
3302348	G	A	synonymous variant	*fadD29*	*Rv2950c*	c.108C>T	p.F36F	long-chain-fatty-acid--AMP ligase FadD29		108	1860	36	619	Low IL1B	6C4
3336646	T	A	intergenic region	*Rv2980-ddlA*	*Rv2980-Rv2981c*	n.3336646T>A	--	--	--	--	--	--		Low IL1B	6C4
3342743	G	A	synonymous variant	*mutT1*	*Rv2985*	c.579G>A	p.Q193Q	8-oxo-dGTP diphosphatase		579	954	193	317	Low IL1B	6C4
3386945	G	A	intergenic region	*Rv3027c-fixB*	*Rv3027c-Rv3028c*	n.3386945G>A	--	--	--	--	--	--		Low IL1B	6C4
3402406	G	C	missense variant	*serB2*	*Rv3042c*	c.757C>G	p.P253A	phosphoserine phosphatase SerB		757	1230	253	409	Low IL1B	6C4
3415180	ACACCTAGGGGGTGG	A	intergenic region	*nrdH-Rv3054c*	*Rv3053c-Rv3054c*	n.3415181 3415194delCACCTAGGGGGTGG	--	--	--	--	--	--		Low IL1B	6C4
3444966	GGGCACCGACGGCC	G	frameshift variant	*pknK*	*Rv3080c*	c.1010 1022delGGCCGTCGGTGCC	p.R337fs	serine/threonine-protein kinase PknK		1022	3333	337	1110	Low IL1B	6C4
3453200	C	A	synonymous variant	*Rv3087*	*Rv3087*	c.276C>A	p.A92A	diacylglycerol O-acyltransferase		276	1419	92	472	Low IL1B	6C4
3511700	A	G	missense variant	*nuoA*	*Rv3145*	c.19A>G	p.I7V	NADH-quinone oxidoreductase subunit A		19	387	7	128	Low IL1B	6C4
3513664	C	T	synonymous variant	*nuoD*	*Rv3148*	c.327C>T	p.A109A	NADH-quinone oxidoreductase subunit D		327	1323	109	440	Low IL1B	6C4
3558749	G	T	intergenic region	*Rv3191c-metU*	*Rv3191c-Rvnt38*	n.3558749G>T	--	--	--	--	--	--		Low IL1B	6C4
3566630	C	T	synonymous variant	*Rv3196*	*Rv3196*	c.843C>T	p.L281L	hypothetical protein		843	900	281	299	Low IL1B	6C4
3598138	C	T	synonymous variant	*rshA*	*Rv3221A*	c.219G>A	p.K73K	anti-sigma factor RshA		219	306	73	101	Low IL1B	6C4
3690255	G	A	synonymous variant	*lpdA*	*Rv3303c*	c.684C>T	p.V228V	NAD(P)H quinone reductase LpdA		684	1482	228	493	Low IL1B	6C4

3700783	G	A	synonymous variant	*Rv3312A*	*Rv3312A*	c.234C>T	p.P78P	pilin	234	312	78	103	Low IL1B	6C4
3722702	G	C	synonymous variant	*trpS*	*Rv3336c*	c.930C>G	p.L310L	tryptophan--tRNA ligase	930	1011	310	336	Low IL1B	6C4
3742991	AG	A	intergenic region	*PE_PGRS50-Rv3346c*	*Rv3345c-Rv3346c*	n.3742992delG	--	--	--	--	--	--	Low IL1B	6C4
3871647	C	T	missense variant	*eccB4*	*Rv3450c*	c.850G>A	p.V284I	ESX-4 secretion system protein EccB4	850	1413	284	470	Low IL1B	6C4
3889558	T	C	missense variant	*Rv3472*	*Rv3472*	c.197T>C	p.V66A	hypothetical protein	197	507	66	168	Low IL1B	6C4
3966303	A	C	missense variant	*Rv3529c*	*Rv3529c*	c.736T>G	p.Y246D	hypothetical protein	736	1155	246	384	Low IL1B	6C4
3972735	G	A	synonymous variant	*hsaF*	*Rv3534c*	c.858C>T	p.S286S	4-hydroxy-2-oxovalerate aldolase	858	1041	286	346	Low IL1B	6C4
4026073	G	A	missense variant	*lpqE*	*Rv3584*	c.244G>A	p.V82I	lipoprotein LpqE	244	549	82	182	Low IL1B	6C4
4034133	G	C	missense variant	*TB11.2*	*Rv3592*	c.77G>C	p.R26P	heme-degrading monooxygenase	77	318	26	105	Low IL1B	6C4
4068928	G	A	synonymous variant	*Rv3629c*	*Rv3629c*	c.127C>T	p.L43L	integral membrane protein	127	1098	43	365	Low IL1B	6C4
4098669	G	A	synonymous variant	*Rv3660c*	*Rv3660c*	c.480C>T	p.G160G	hypothetical protein	480	1053	160	350	Low IL1B	6C4
4103228	G	A	missense variant	*dppD*	*Rv3663c*	c.451C>T	p.P151S	dipeptide ABC transporter ATP-binding protein DppD	451	1647	151	548	Low IL1B	6C4
4207243	A	C	missense variant	*Rv3762c*	*Rv3762c*	c.1634T>G	p.F545C	hydrolase	1634	1881	545	626	Low IL1B	6C4
4264734	G	A	synonymous variant	*fbpD*	*Rv3803c*	c.729C>T	p.I243I	MPT51/MPB51 antigen	729	900	243	299	Low IL1B	6C4
4288212	C	T	intergenic region	*Rv3822-mmpL8*	*Rv3822-Rv3823c*	n.4288212C>T	--	--	--	--	--	--	Low IL1B	6C4
4320250	G	T	intergenic region	*Rv3845-sodA*	*Rv3845-Rv3846*	n.4320250G>T	--	--	--	--	--	--	Low IL1B	6C4
4323931	G	A	intergenic region	*espR-Rv3850*	*Rv3849-Rv3850*	n.4323931G>A	--	--	--	--	--	--	Low IL1B	6C4
4335281	C	G	synonymous variant	*gltB*	*Rv3859c*	c.801G>C	p.A267A	glutamate synthase large subunit	801	4584	267	1527	Low IL1B	6C4
4337005	A	C	missense variant	*Rv3860*	*Rv3860*	c.229A>C	p.T77P	hypothetical protein	229	1173	77	390	Low IL1B	6C4
4345420	G	T	missense variant	*eccB1*	*Rv3869*	c.382G>T	p.V128F	ESX-1 secretion system protein EccB	382	1443	128	480	Low IL1B	6C4
4374184	A	G	synonymous variant	*esxD*	*Rv3891c*	c.189T>C	p.N63N	ESAT-6 like protein EsxD	189	324	63	107	Low IL1B	6C4
4398141	G	A	synonymous variant	*Rv3910*	*Rv3910*	c.1545G>A	p.S515S	peptidoglycan biosynthesis protein	1545	3555	515	1184	Low IL1B	6C4
4404694	G	A	synonymous variant	*Rv3916c*	*Rv3916c*	c.474C>T	p.G158G	hypothetical protein	474	735	158	244	Low IL1B	6C4

Supplementary Table 5. List of oligonucleotide sequences and information.

**Oligonucleotides (name and sequence 5'-3')**	**Provider**	**Catalogue number or reference**
Forward primer Mouse IL-1β: ACCTTCCAGGATGAGGACATGA	Invitrogen	
Reverse primer Mouse IL-1β: ACGTCACACACCAGCAGGTTA	Invitrogen	
Forward primer Mouse IFN-β: GCACTGGGTGGAATGAGACT	Invitrogen	
Primer Mouse IFN-β Reverse: AGTGGAGAGCAGTTGAGGACA	Invitrogen	
Primer Mouse TNF-α Forward: GCCACCACGTCTTCTGTCT	Invitrogen	
Reverse primer Mouse TNF-α : TGAGGGTCTGGGCCATAGAAC	Invitrogen	
Forward primer Mouse IL-10: ATTTGAATTCCCTGGGTGAGAAG	Invitrogen	
Reverse primer Mouse IL-10: CACAGGGGAGAAATCGATGACA	Invitrogen	
Forward primer Mouse Ubiquitin: TGGCTATTAATTATTGGGTCTGCAT	Invitrogen	
Reverse primer Mouse Ubiquitin: GCAAGTGGCTAGAGTGCAGAGTAA	Invitrogen	
TaqMan Primer-Probes Mouse CCL2	Applied Biosystems	Mm00441242_m1
TaqMan Primer-Probes Mouse CXCL10	Applied Biosystems	Mm99999072_m1
TaqMan Primer-Probes Mouse HPRT	Applied Biosystems	Mm00446968_m1
MTBC genotyping	Applied Biosystems	Stucki et al., 2012*

*Stucki D, *et al*. Two new rapid SNP-typing methods for classifying Mycobacterium tuberculosis complex into the main phylogenetic lineages. *PloS one* **7**, e41253 (2012).
