# Supplementary material for: Mycobacterium tuberculosis associated with severe tuberculosis evades cytosolic surveillance systems and modulates IL-1β production
Source: Nat Commun. 2020 Apr 23;11:1949. doi: 10.1038/s41467-020-15832-6 (PMC7181847; doi:10.1038/s41467-020-15832-6)
Supplement: Supplementary file 3 — Reporting Summary [file 41467_2020_15832_MOESM3_ESM.pdf]

nature research

Corresponding author(s): Margarida Saraiva

Last updated by author(s): 2020/02/20

Reporting Summary

Nature Research wishes to improve the reproducibility of the work that we publish. This form provides structure for consistency and transparency in reporting. For further information on Nature Research policies, see Authors & Referees and the Editorial Policy Checklist .

Statistics

For all statistical analyses, confirm that the following items are present in the figure legend, table legend, main text, or Methods section.

n/a	Confirmed	
☐	☒	The exact sample size (*n*) for each experimental group/condition, given as a discrete number and unit of measurement
☐	☒	A statement on whether measurements were taken from distinct samples or whether the same sample was measured repeatedly
☐	☒	The statistical test(s) used AND whether they are one- or two-sided<br>*Only common tests should be described solely by name; describe more complex techniques in the Methods section.*
☒	☐	A description of all covariates tested
☐	☒	A description of any assumptions or corrections, such as tests of normality and adjustment for multiple comparisons
☐	☒	A full description of the statistical parameters including central tendency (e.g. means) or other basic estimates (e.g. regression coefficient) AND variation (e.g. standard deviation) or associated estimates of uncertainty (e.g. confidence intervals)
☐	☒	For null hypothesis testing, the test statistic (e.g. *F*, *t*, *r*) with confidence intervals, effect sizes, degrees of freedom and *P* value noted<br>*Give P values as exact values whenever suitable.*
☒	☐	For Bayesian analysis, information on the choice of priors and Markov chain Monte Carlo settings
☒	☐	For hierarchical and complex designs, identification of the appropriate level for tests and full reporting of outcomes
☐	☒	Estimates of effect sizes (e.g. Cohen's *d*, Pearson's *r*), indicating how they were calculated

Our web collection on statistics for biologists contains articles on many of the points above.

Software and code

Policy information about availability of computer code

Data collection	Bio-Rad CFX Manager™ 3.1, IN Cell Analyzer 2000 acquisition software (v4.5), Torrent Suite software (v5.10)
Data analysis	CLUMPP, DISTRUCT, Bio-Rad CFX Manager™ 3.1, Trimmomatic v0.38, FastQC v0.11.7, MultiQC v1.0, SAMtools v1.3.1, Mark Duplicates v2.18.14, Pilon v1.22, bcftools v1.3.1, SnpEff v4.3t, RaxML v7.2.8 , iTOL v4.3.3, Treemer, Fiji, R v3.5.1, edgeR v3.24.1, limma v3.38.3, ReactomePA v1.26.0, GraphPad Prism v 8.1.0, Beanplot

For manuscripts utilizing custom algorithms or software that are central to the research but not yet described in published literature, software must be made available to editors/reviewers. We strongly encourage code deposition in a community repository (e.g. GitHub). See the Nature Research guidelines for submitting code & software for further information.

Data

Policy information about availability of data

All manuscripts must include a data availability statement. This statement should provide the following information, where applicable:

- Accession codes, unique identifiers, or web links for publicly available datasets
- A list of figures that have associated raw data
- A description of any restrictions on data availability

Generated data from RNA-Seq are deposited on NCBI GEO database accession code GSE138580

Field-specific reporting

Please select the one below that is the best fit for your research. If you are not sure, read the appropriate sections before making your selection.

Life sciences study design

All studies must disclose on these points even when the disclosure is negative.

Sample size	No calculation was performed, but based on previous experiments sample sizes were large enough to detect the effects of interest holding biological significance.
Data exclusions	No data was excluded from the analysis.
Replication	At least triplicates were included, with most experiments performed at least twice using independent cell cultures and infections. All replications provided concordant results.
Randomization	No randomization was performed, as all available patients within the inclusion criteria were included and as for each in vitro experiment involving cell cultures the same source was used. Thus, randomization was not relevant for our study.
Blinding	Digital images of plain chest radiographs were blind-graded by two independent clinicians. Disagreements between the two clinicians were resolved through a consensus assessment by a third reader. For other experiments blinding was not possible, as the investigator performing the experiments were the ones analysing them.

Reporting for specific materials, systems and methods

We require information from authors about some types of materials, experimental systems and methods used in many studies. Here, indicate whether each material, system or method listed is relevant to your study. If you are not sure if a list item applies to your research, read the appropriate section before selecting a response.

Materials & experimental systems

n/a	Involved in the study	
☐	☒	Antibodies
☐	☒	Eukaryotic cell lines
☒	☐	Palaeontology
☐	☒	Animals and other organisms
☐	☒	Human research participants
☒	☐	Clinical data

Methods

n/a	Involved in the study	
☒	☐	ChIP-seq
☒	☐	Flow cytometry
☒	☐	MRI-based neuroimaging

Antibodies

Antibodies used	Mouse IFNb capture antibody (BioLegend; cat# 519202) and detection antibody (BioLegend; cat# 508105); ELISA mouse IL-1β Kit (ThermoFisher Scientific- Invitrogen; 88-7013-88); ELISA mouse TNF-α Kit (ThermoFisher Scientific- Invitrogen; 88-7324-88); ProcartaPlex Human IL-1β (EPX01A-10224-901), IL-12/ IL-12p40 (EPX01A-12090-901) and IL-10 (EPX01A-10215-901) simplex kits (all ThermoFisher Scientific - Invitrogen).
Validation	All antibodies used are commercially available and were validated by the manufacturer.

Eukaryotic cell lines

Policy information about cell lines

Cell line source(s)	THP1-ASC-GFP (InvivoGen; cat# thp-ascgfp)
Authentication	Commercially available. Cell lines were not further authenticated.
Mycoplasma contamination	Not tested
Commonly misidentified lines (See ICLAC register)	No commonly misidentified lines are used in the study

Animals and other organisms

Policy information about studies involving animals; ARRIVE guidelines recommended for reporting animal research

Laboratory animals	Eight-to-12-week-old male or female mice; Strains C57BL/6 WT, TLR2-/-, TLR4-/-, IFNAR-/-, AIM2-/-. Used for the generation of mouse bone marrow derived macrophages.
Wild animals	No wild animals were used

Field-collected samples	No field collected samples were used.
Ethics oversight	All experiments were performed in strict accordance with recommendation of European Union Directive 2010/63/EU and previously approved by Portuguese National Authority for Animal Health - Direção Geral de Alimentação e Veterinária(DGAV)

Note that full information on the approval of the study protocol must also be provided in the manuscript.

Human research participants

Policy information about studies involving human research participants

Population characteristics	the population is fully described in: Bastos, H.N. et al. A Prediction Rule to Stratify Mortality Risk of Patients with Pulmonary Tuberculosis. PloS one 11, e0162797, doi:10.1371/journal.pone.0162797<br>1371/journal.pone.0162797
Recruitment	the blood for PBMC isolation was obtained from participants enrolled at a TB clinics in Porto. During recruitment it was ensured that no self bias was introduced. For example, IGRA tests were taken into consideration.
Ethics oversight	The study protocool leading to PBMC isolation was approved by the Health Ethics Committees of the CHSJ (approval number 109-11), the North Health Region Administration (approval number 71-2014) and the Portuguese Data Protection Authority (approval number 12174-2011).

Note that full information on the approval of the study protocol must also be provided in the manuscript.
